# Rapid and On-Scene Chemical Identification of Intact Explosives with Portable Near-Infrared Spectroscopy and Multivariate Data Analysis

**DOI:** 10.3390/s23083804

**Published:** 2023-04-07

**Authors:** Irene M. van Damme, Pol Mestres-Fitó, Henk-Jan Ramaker, Annemieke W. C. Hulsbergen, Antoine E. D. M. van der Heijden, Ruben F. Kranenburg, Arian C. van Asten

**Affiliations:** 1 Van ’t Hoff Institute for Molecular Sciences, University of Amsterdam, Science Park 904, 1098 XH Amsterdam, The Netherlands; 2 Netherlands Forensic Institute (NFI), Laan van Ypenburg 6, 2497 GB Den Haag, The Netherlands; 3 TIPb, Koningin Wilhelminaplein 30, 1062 KR Amsterdam, The Netherlands; 4 TNO Defence, Safety and Security, Department Energetic Materials, Ypenburgse Boslaan 2, 2496 ZA The Hague, The Netherlands; 5 Dutch National Police, Unit Amsterdam, Forensic Laboratory, Kabelweg 25, 1014 BA Amsterdam, The Netherlands; 6 Co van Ledden Hulsebosch Center (CLHC), Amsterdam Center for Forensic Science and Medicine, Science Park 904, 1098 XH Amsterdam, The Netherlands

**Keywords:** explosives, NIR, chemical identification, chemometrics, on-scene analysis, portable analysis, forensic science

## Abstract

There is an ongoing forensic and security need for rapid, on-scene, easy-to-use, non-invasive chemical identification of intact energetic materials at pre-explosion crime scenes. Recent technological advances in instrument miniaturization, wireless transfer and cloud storage of digital data, and multivariate data analysis have created new and very promising options for the use of near-infrared (NIR) spectroscopy in forensic science. This study shows that in addition to drugs of abuse, portable NIR spectroscopy with multivariate data analysis also offers excellent opportunities to identify intact energetic materials and mixtures. NIR is able to characterize a broad range of chemicals of interest in forensic explosive investigations, covering both organic and inorganic compounds. NIR characterization of actual forensic casework samples convincingly shows that this technique can handle the chemical diversity encountered in forensic explosive investigations. The detailed chemical information contained in the 1350–2550 nm NIR reflectance spectrum allows for correct compound identification within a given class of energetic materials, including nitro-aromatics, nitro-amines, nitrate esters, and peroxides. In addition, the detailed characterization of mixtures of energetic materials, such as plastic formulations containing PETN (pentaerythritol tetranitrate) and RDX (trinitro triazinane), is feasible. The results presented illustrate that the NIR spectra of energetic compounds and mixtures are sufficiently selective to prevent false-positive results for a broad range of food-related products, household chemicals, raw materials used for the production of home-made explosives, drugs of abuse, and products that are sometimes used to create hoax improvised explosive devices. However, for frequently encountered pyrotechnic mixtures, such as black powder, flash powder, and smokeless powder, and some basic inorganic raw materials, the application of NIR spectroscopy remains challenging. Another challenge is presented by casework samples of contaminated, aged, and degraded energetic materials or poor-quality HMEs (home-made explosives), for which the spectral signature deviates significantly from the reference spectra, potentially leading to false-negative outcomes.

## 1. Introduction

On-scene identification of intact explosives must be fast and accurate to ensure the safety of people on the scene and those handling the evidence. Underestimating the threat of a material leads to uninformed decisions that could cause death or severe injuries, whereas overestimating the threat could enable criminal exploitation of hoax materials and generally results in a waste of resources. In terms of safety, efficiency, and cost, not having to send samples to the laboratory for analysis is generally beneficial. However, such a development requires a safe and reliable technique that ideally yields admissible evidence on site. The broad chemical range of explosive compounds poses a challenge in this regard. Investigators cannot visually determine whether an energetic material is of an organic or inorganic nature, and whether it is a mixture rather than a pure compound. Therefore, suitable portable technology must be able to confidently identify each explosive substance that could potentially be present.

Traditionally, colorimetric tests have been used for on-scene explosives detection. These tests detect classes of compounds and can indicate the possible presence of an explosive, but lack selectivity and are typically unable to identify an explosive within a class [1]. They also require manual sampling and sample handling, which introduces additional risk. Moreover, a single colorimetric test covers only a sub-range of explosives and the interpretation of the color formation is, to some degree, subjective. Although some of these drawbacks can be mitigated by using microfluidic paper-based analytical devices (μPADs) [2,3], alternative approaches have been proposed for on-scene identification of intact energetic materials. A widely used method for rapid explosives detection is ion mobility spectrometry (IMS) [4]. Given its high sensitivity, aviation security mostly applies this technique to detect trace amounts on luggage. IMS is less favorable for the identification of bulk amounts, considering the potential overloading of the instrument leading to false-positive results in subsequent analyses [5]. Recent advances in portable mass spectrometry (MS) provide high selectivity and sensitivity on site, but this approach is relatively expensive and technologically challenging [6]. The required vacuum for MS provides challenges and hampers miniaturization. Advances in fluorescence quenching sensor arrays enable sensitive on-site detection of explosive vapors and liquids [7,8,9,10,11,12,13,14]. These arrays proved successful in the detection of nitro-based explosives, owing to fluorescence quenching by electron-withdrawing nitro groups [10]. Differential binding to fluorophores or to quantum dots in proximity of fluorescent nanoparticles allows selective differentiation between nitro-based explosives [11,12,13,14]. While these sensor arrays do not cover the entire range of explosives and do not provide compound-specific identification, their reported sensitivity, low cost, and simplicity are promising for practical field applications. Interesting developments are also being reported in the electrochemical detection of explosives [15,16,17]. Molecularly imprinted polymers are utilized to achieve high selectivity in targeted electrochemical analysis [18]. However, for bulk analysis of unknown energetic materials, a single, non-invasive analysis covering the entire chemical range of explosives is preferred.

Spectroscopic techniques are suitable for bulk samples, provide highly characteristic chemical information, and have the advantage of requiring minimal to no sample preparation. Raman spectroscopy is non-invasive, but its signal is obscured by fluorescent samples [19]. Moreover, Raman laser sources have sufficient power to potentially burn samples, compromising safety on the scene and risking evidence loss [20]. Interestingly, using portable Fourier-transform infrared spectroscopy (FT-IR) does not entail such risks but requires sampling and more extensive sample preparation [19]. Near-infrared (NIR) spectroscopy is non-invasive and not affected by matrix fluorescence, and strongly reduces the risk of ignition [19,20,21]. Additionally, NIR analyzers are relatively cheap and can be miniaturized, and, therefore, have the potential to be routinely employed in the field [21]. NIR spectra alone are not informative enough for structural elucidation, since the bands in the NIR region (780–2500 nm) are weak and result from complex combined vibrations and overtone absorptions [22,23]. This lack of signal interpretability can be overcome by pre-processing the raw data and applying chemometric methods (multivariate data analysis) to extract informative features from the data [21,23].

NIR combined with multivariate data analysis has been applied in the last decade for the analysis of intact explosives [24,25,26,27,28,29,30,31,32,33]. Interestingly, the approach proved successful for explosive trace detection on various substrates [24,25,26,27]. Other studies demonstrated accurate composition analysis of explosives using NIR [28,29,30]. The miniaturization of NIR analyzers has rapidly progressed over the years and facilitates new practical applications, such as the detection of bottled explosive liquids in airports [31,34,35]. The recent literature demonstrates that handheld NIR analyzers can identify subsets of explosives and precursors at high correct classification rates [32,33]. However, the use of portable NIR for real-time decision-making at the incident scene requires the technique to be highly selective across the full range of energetic materials potentially present.

The aim of this research is to develop a portable analysis platform capable of identifying a broad range of intact compounds and materials that are of relevance in forensic explosives investigation with high confidence (i.e., with a very low risk of false-positive or false-negative outcomes). Our approach is based on portable NIR and tailor-made multivariate data analysis strategies, requires minimal sample handing, and provides a sample assessment in seconds with a measurement that can be conducted with minimal instructions and requires no chemical expertise. A powerful, portable FT-NIR analyzer from Si-Ware was employed to cover a broad wavelength range of 1350–2550 nm. This analyzer is equipped with a proprietary MEMS (microelectromechanical systems) sensor. Previously, this setup was successfully applied to identify and characterize an extensive set of frequently encountered drugs of abuse. High accuracy was achieved using a multi-stage chemometric model, which included a linear discrimination analysis (LDA) component and a net analyte signal (NAS) model [36,37,38,39,40]. To address the chemical diversity in the field of forensic explosive investigations, the data analysis strategy was further refined through a three-stage approach. A special explosives matrix was constructed, consisting of pure compound spectra of organic and inorganic energetic compounds encountered in forensic explosives casework. Chemical selectivity was tested by comparing the NIR spectra of organic explosives with very similar molecular structures, e.g., ETN (erythritol tetranitrate) vs. PETN and RDX vs. HMX (tetranitro tetrazocane). Furthermore, the capability of multivariate data analysis methods to correctly characterize the composite NIR spectra of mixture formulations was investigated. To this end, binary RDX/PETN mixtures were prepared and analyzed along with a set of plastic explosives of the C4 and Semtex type. The risk of false-positive outcomes was mapped by analyzing a large set of chemicals and products (food stuffs, household chemicals, hoax materials, drugs of abuse, and raw materials) that could be mistakenly considered as explosives during an investigation. In addition, a set of forensic casework samples from the Netherlands Forensic Institute (NFI) was analyzed and the NIR model output was compared to results of the more elaborate analyses conducted by forensic experts to assess the false-negative rate. Overall, the results presented in this work illustrate the substantial added value that portable NIR could bring for the rapid and robust chemical identification of intact explosives.

## 2. Materials and Methods

### 2.1. Chemical Standards

Sodium chlorate (NaClO_3_, ACS Reagent, ≥99.0%), sodium perchlorate (NaClO_4_, 99%), and potassium perchlorate (KClO_4_, ACS Reagent, ≥99.0%) were purchased from Sigma-Aldrich (St. Louis, MO, USA). In addition, KClO_4_ (anhydrous, ACS, 99.0–100.5%), KClO_4_ (99%), and KClO_3_ (ACS, 99.0%) standards were obtained from abcr GmbH (Karlsruhe, Germany) and KClO_4_ (anhydrous, ACS, 99.0–100.5%) and KClO_4_ (99%) were acquired from ThermoFisher GmbH (Kandel, Germany). Additional batches of KClO_3_ (≥99.7%) were obtained from Carl Roth GmbH (Karlsruhe, Germany) and Boom B.V. (AnalaR NORMAPUR, Meppel, The Netherlands). Finally, potassium nitrate (KNO_3_) was acquired from Janssen Chimica (Beerse, Belgium).

### 2.2. Test Samples

Because of the risks associated with energetic materials, standards of explosive compounds are only commercially available as low concentration solutions in organic solvents. Such solutions cannot be used to establish reflectance NIR spectra of the compounds of interest. Hence, most reference spectra, with the exception of some raw material salts, were obtained from samples made available by the scientists of the Department of Energetic Materials of TNO Defence, Safety and Security and the forensic explosives experts of the NFI. These samples were synthesized in-house, were part of a collection of professional and military grade explosive materials, or originated from forensic casework. Although these materials are less controlled than standards and chemicals ordered from commercial suppliers, relatively pure reference samples were selected on the basis of in-house chemical analysis.

A total of 48 pure explosive samples and mixtures were analyzed with portable NIR at TNO: 1 AN (ammonium nitrate) sample, 2 ETN samples, 1 HMTD (hexamethylene triperoxide diamine) sample, 6 HMX (tetranitro tetrazocane) samples, 4 NC (nitrocellulose) samples, 1 PA (picric acid) sample, 2 PETN (pentaerythritol tetranitrate) samples, 13 RDX (trinitro triazinane) samples, 2 TATP (triacetone triperoxide) samples, 1 tetryl (trinitrophenyl methylnitramine) sample, 3 TNT (trinitro toluene) samples, 5 Semtex (RDX, PETN, and additives) samples, 2 C4 (RDX with additives) samples, 3 double base powder (DBP, containing nitrocellulose and nitroglycerine) samples, and 2 gunpowder (black powder) samples. In addition, TNO scientists prepared binary mixtures of RDX and PETN in various ratios (in total 11 samples, from 100 wt% RDX (TL 144/96) in steps of 10 wt% to 100 wt% PETN (TL 977/18)). Information on the explosive material samples from TNO is provided in Table S1 of the Supplemental Information.

In total, 74 samples were provided by the NFI. This set consisted of 1 UN (urea nitrate) sample prepared at the NFI, 1 HMTD sample prepared at the NFI, 2 ETN samples prepared at TNO, 1 AN (≥99.0%) sample from Sigma-Aldrich (St. Louis, MO, USA), and 69 samples originating from casework between 2005 and 2022 (3 TATP samples, 8 HMTD samples, 6 TATP/HMTD mixtures (2 with aluminum), 8 PETN samples, 4 RDX samples, 5 TNT samples, 10 tetryl samples, 16 AN samples (6 with aluminum), 2 AN-based plastic emulsions, 6 NC samples and 2 flash powder (mixture of potassium perchlorate and aluminum) samples, and 1 sample also contained sulfur). Information on the case samples from the NFI is criminal case sensitive and cannot be shared without prior approval of the Dutch Public Prosecution Office.

Smokeless powder (SP) reference materials were obtained from the National Center for Forensic Science (NCFS, Orlando, FL, USA). Several SP samples were screened with NIR in this study (SRN 300, 305, 306, 308, 314, 315, 321, 326, 329, 330, 331, 332, 334, 355–363, 367, 368, 370, 377, 379, 388, 392, 394, 395, and 396). More information on these samples can be found using the SRN code in the on-line NCFS smokeless powders database (https://www.ilrc.ucf.edu/powders/, (accessed on 6 March 2023)).

### 2.3. Negative Samples

To test the selectivity of the NIR spectrometer and investigate potential false-positive outcomes, a diverse ‘negative’ sample set was created consisting of various common substances. Such substances could be mistaken for an explosive formulation or could intentionally be used to mimic an improvised explosive device (a so-called hoax). This set included household chemicals (agar-agar, baking powder, baking soda, coffee creamer, pain killer powders, fondant, powdered sugar, table sugar, vetsin (monosodium glutamate), washing detergent, wheat flower, table salt, Play-Doh clay, and corn starch), raw materials used for explosive synthesis (erythritol, xylitol), and illicit drugs and associated adulterants (3-methylmethcathinone (3-MMC), amphetamine, aspirin, benzocaine, boric acid, caffeine, cocaine HCl and base, diltiazem, phenacetine, flunitrazepam, gamma-hydroxybutyric acid (GHB), brown and white heroin, ketamine, levamisole, lidocaine, mannitol, 3,4-methylenedioxy-methamphetamine (MDMA), mephedrone, methamphetamine, oxazepam, paracetamol, phenacetine, procaine, promethazine, sildenafil (Viagra), tetrahydrocannabinol (THC), vitamin C, and starch). All household and foodstuff substances were obtained from local Dutch grocery stores, drug-related substances were provided by the Amsterdam Police Laboratory.

### 2.4. NIR Measurements

**Figure 1:**
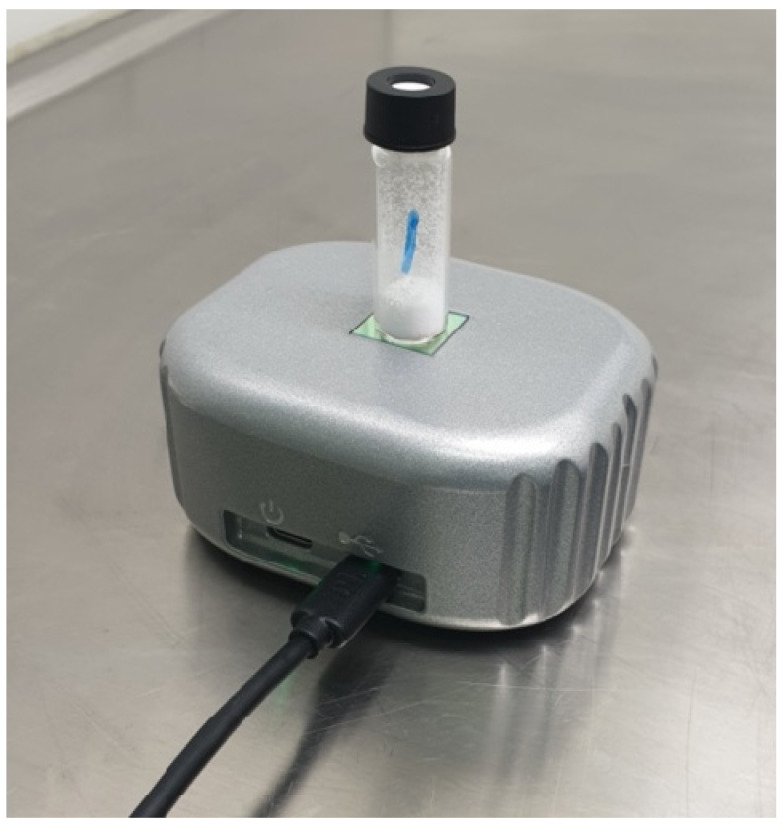
Positioning of a borosilicate glass vial with an energetic material on the scanner of the portable NIR analyzer.

NIR characterization of the samples made available by TNO and NFI was performed on-site to prevent the transportation of energetic material and minimize the risks of sample loss and accidental activation. NIR measurements were conducted in diffuse reflectance mode by placing a regular, NIR transparent borosilicate glass vial (4 mL volume, 15 mm diameter) on the NIR analyzer, as is illustrated in Figure 1. The use of smaller vials is not recommended because of incomplete coverage of the NIR sensor surface. Roughly 0.5 g of sample material was added to the glass vials to minimize the effects of an unexpected explosion/deflagration while ensuring a sufficient layer thickness (>0.5 cm) for a high-quality reflectance NIR measurement. NIR spectra were recorded in the wavelength range of 1350–2550 nm using a portable spectrometer (10 × 8 × 4.5 cm, 550 g) equipped with an FT-NIR MEMS sensor from Si-Ware (Cairo, Egypt). According to specifications, this sensor has a resolution of 16 nm at 1550 nm. For each sample, 5 NIR spectra were recorded, where the vial was shaken and repositioned on the scanner in between measurements to capture spectral variation as function of sample position and powder arrangement. For a selection of the samples, 3 spectra were also recorded for a fixed vial and sample position to capture the intrinsic measurement variation of the NIR sensor. A single NIR scan was typically completed within a few seconds, after which the spectrum was recorded and displayed using the NeoSpectra SpectroMOST2 software (version 2.0.10, Si-Ware Systems, 3, Khaled Ibn Al-Waleed St. Sheraton, Heliopolis, Cairo 11361, Egypt) running on a laptop connected to the NIR analyzer through a USB-A to USB-C cable. The SpectroMOST software was also used to control the scanner settings, perform blank measurements using the Fluorilon (PTFE) total reflector, and save the scan data. A single scan consists of absorbance values at 257 discrete wavelengths in the 1350–2550 nm range. The absorbance value at a given wavelength is calculated as the negative logarithm of the ratio of the detected ‘light’ intensity reflected from the sample and the overall intensity of the source radiation at that wavelength (as measured with the Fluorilon reflector). The NeoSpectra data files (15 kB in size per scan) were converted to csv files using a home-made MATLAB (2020b update 5, MathWorks, Natick, MA, USA) script.

### 2.5. Data Pre-Processing and (Multivariate) Analysis

Excel (version 2301, Microsoft, Redmond, WA, USA) and Unscrambler X (version 10.5.1, Camo Analytics, Oslo, Norway) were used for data inspection, pre-processing, and visualization to produce the raw and processed NIR spectra shown in this paper. Data pre-processing to remove baseline shifts and minimize non-specific measurement variation included sum normalization, standard normal variate (SNV, subtraction of the average signal over the entire wavelength range and division by the associated standard deviation), and taking the first derivative of the measured spectrum.

Multivariate data analysis (Principal Component Analysis—PCA, Partial Least Squares—PLS, and Linear Discriminant Analysis—LDA) was conducted using Unscrambler X. Standard Unscrambler settings were used when applying these data analysis methods. SNV (Standard Normal Variate) data pre-processing was consistently applied and for the LDA model for perchlorate discrimination (Figure 10), this was followed by a Savitzky–Golay first derivative algorithm (3rd order polynomial, symmetric kernel of 5 data points). All data were mean-centered but not auto-scaled nor selectively weighted prior to modeling. PCA analysis was conducted using the Singular Value Decomposition (SVD) algorithm and PLS was performed with the Kernel PLS algorithm both for a maximum of 7 components. For PCA and PLS, cross-validation was conducted with 20 segments containing 2 or 3 randomly selected spectra from the standard data set. LDA was performed with the PCA scores for the first 7 principal components using the linear model option and applying equal prior probabilities.

**Figure 2:**
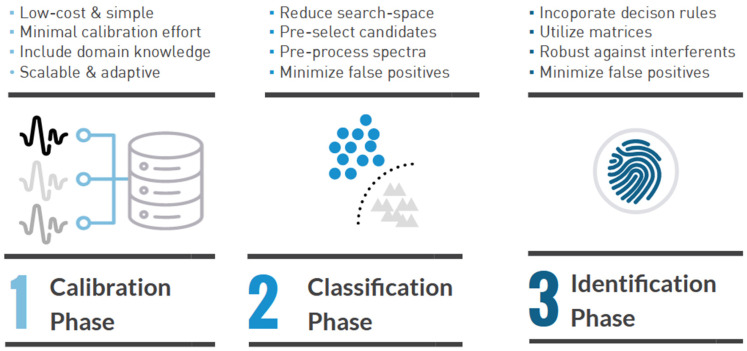
Diagram illustrating the various phases in the applied scheme for NIR-based identification of intact energetic materials in forensic explosives casework using the three-stage TIPb chemometric model.

In addition, a more advanced, tailor-made, 3-stage chemometric model was developed by TIPb for the NIR characterization of forensic explosives casework samples. This multi-stage model is illustrated in Figure 2. During the first stage of the model, the near-infrared sensor is calibrated by using a so-called explosives matrix. This matrix is defined by all possible chemical components that explosive materials can be made of and its contents are based on casework experience and knowledge from forensic experts. An overview of the explosives matrix and its components is provided in Table S2 of the Supplemental Information.

The next phase of the TIPb chemometric model consists of the construction of an LDA classification model. This model makes use of the spectral library that is represented by the explosives reference matrix and acts as a filter to pre-select candidates (or chemical components) and forward these to the identification phase. By pre-selecting chemical components, the search-space of possible combinations of matrix components is further reduced while minimizing the false-positive rate.

In phase 3 of the TIPb chemometric model, the identification of unknown samples is performed. Based on the Net Analyte Signal (NAS) approach, an unknown spectrum is reconstructed from the pre-selection of matrix components. The predicted spectrum by the identification model is compared to the unknown measurement by means of a similarity measurement. The identification is significant if the similarity between the prediction and true measurement exceeds 0.8 at a maximum score of 1.0. The identification phase also allows for the inclusion of decision rules, for instance to rule out unlikely combinations of matrix components. The NAS model is not only capable of leveraging the information represented by the explosive matrix, but is, to a certain, extent also robust to unknown interferents that have not been included in the explosives reference matrix.

## 3. Results

### 3.1. Data Preprocessing of NIR Spectra of Energetic Materials

**Figure 3:**
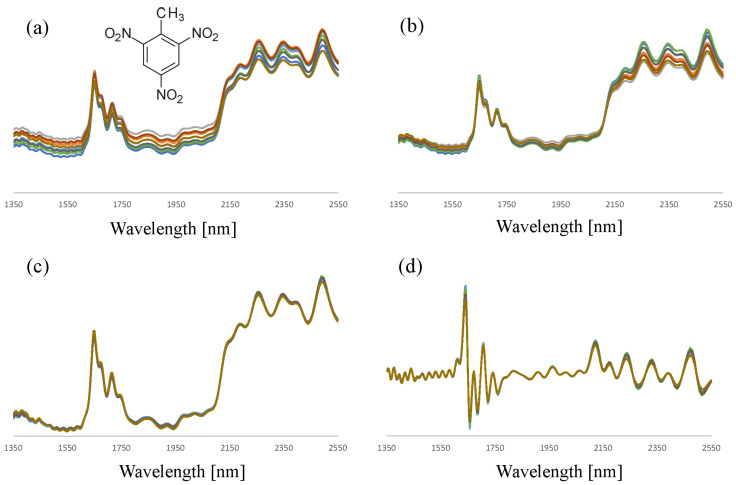
NIR spectra (n = 10, 2 samples) of TNT as measured with the NIR analyzer (**a**) and after sum normalization (**b**), SNV (**c**), and taking the first derivative (**d**) of the absorbance.

When employing diffuse reflection NIR spectroscopy for the characterization of solid materials, data pre-processing is an essential first step for maximizing chemometric model performance. Figure 3 illustrates the spectral variation encountered for two TNT (trinitrotoluene, a high explosive from the nitro-aromatic class) samples and the spectral similarity after applying various data-preprocessing strategies. Consistently, SNV and first derivative processing were found to be very effective in reducing spectral variations for a given compound and thus maximizing the chemical structural information in the NIR spectra. These pre-processing methods have subsequently been applied prior to multivariate data analysis.

### 3.2. NIR Analysis of Organic Explosives

**Figure 4:**
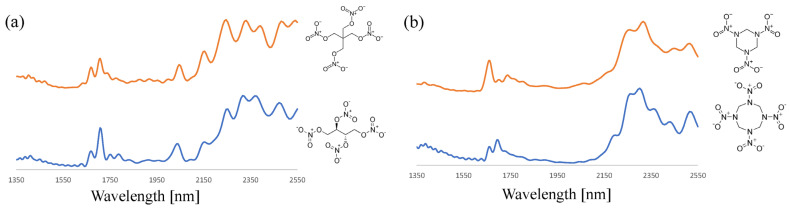
SNV-corrected, average NIR spectra (n = 5) of (**a**) PETN and ETN (orange and blue trace, respectively) and (**b**) RDX and HMX (orange and blue trace, respectively). The offset has been adjusted to facilitate spectrum comparison.

By employing the data preprocessing strategies described above, the NIR analysis of a wide range of organic explosives was undertaken. The NFI- and TNO-based sample set covered nitroaromatics (TNT, PA, and Tetryl), nitro-amines (RDX and HMX), nitrate esters (ETN, PETN, and NC) and peroxides (TATP and HMTD). Figure 4 depicts the SNV-corrected, average spectra of ETN and PETN of the nitrate ester class and RDX and HMX of the nitro-amine class. Although these compound pairs are chemically very similar, the NIR spectra in the 1350–2550 nm range show many spectral features that could be used for differentiation and to identify the correct explosive. Such features are also abundant when zooming in on the 1350–1625 nm part of the spectrum where NIR absorption is less pronounced (Figure S1 of the Supplemental Information). The complex patterns are not instrumental artefacts but were found to be highly reproducible and compound specific.

**Figure 5:**
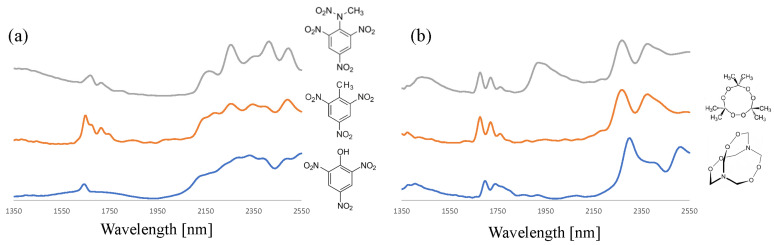
SNV-corrected, average NIR spectra (n = 5) of (**a**) tetryl, TNT, and picric acid (grey, orange, and blue trace, respectively) and (**b**) TATP (wet), TATP (dry), and HMTD (grey, orange, and blue trace, respectively). The offset has been adjusted to facilitate spectrum comparison.

The observed spectra for the most relevant compounds of the nitro-aromatic and peroxide class are shown in Figure 5. Consistently, detailed and reproducible NIR spectra are obtained that are highly characteristic and are sufficiently selective to identify the correct compound from the set of organic explosives. An interesting difference can be noted for the two TATP spectra displayed in Figure 5b. The two additional, pronounced and broad absorption bands at 1450 and 2000 nm relate to the presence of residual water in a TATP sample that was freshly prepared at TNO. This is not unexpected as TATP crystallizes from an aqueous solution and the product is typically washed with water. Additionally, for other explosive compounds, such as nitrocellulose and home-made ETN, water can be present in samples encountered in casework. For chemometric modeling, it is important to include reference spectra that reflect known states (e.g., water of crystallization) and compositions (wet versus dry, see also Table S2 of the Supplemental Information).

**Figure 6:**
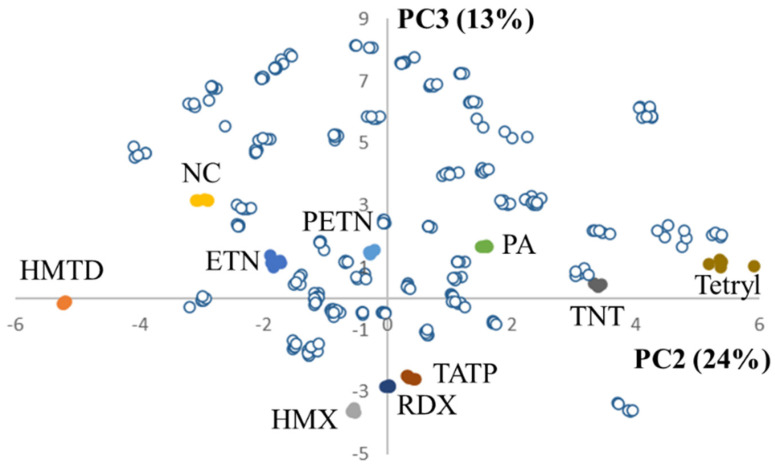
PCA score plot (PC2—explaining 24% of the data variance vs. PC3—explaining 13% of the data variance) of several organic explosives with the negative samples projected in the PCA space (open markers). The PCA model is constructed from a total of 50 NIR reference spectra of 10 organic explosives (excluding the wetted materials), after which a total of 266 spectra of the 51 samples from the ‘negative’ sample set (Section 2.3) were projected in the model.

To assess selectivity, the NIR spectra of a broad range of other chemicals that might be mistaken as or intentionally used to mimic an explosive were acquired, including common household chemicals, food products, raw materials used for the production of explosives, and drugs of abuse and associated adulterants (in forensic casework it can sometimes be unclear whether materials are related to drug or explosives production). A first indication of NIR selectivity was obtained by creating a PCA model for the most frequently occurring organic explosives and projecting the extensive negative set in the model. Figure 6 illustrates that even with an unsupervised multivariate data analysis method like PCA, no overlap occurred for all 51 products in the negative set with any of the organic explosives clusters (the plot of PC1, accounting for 39% of the data variance, vs. PC2 was less compound specific; PC1 possibly represents a source of more generic variation). These results are very promising and further demonstrate the detailed molecular structural information that is contained in the 1350–2550 nm NIR data. As discussed in Section 3.5, such information can be exploited in a more advanced manner using tailor-made supervised data analysis methods. This is demonstrated with the chemometric framework that was specifically developed for NIR-based forensic explosive analysis by TIPb.

### 3.3. NIR Characterization of Mixtures of Organic Explosives

In forensic explosives casework, intact energetic materials can show a high degree of purity (e.g., washed TATP crystals) but can also occur as mixtures. Both HME (home-made explosive), professional engineering, and military explosive formulations can consist of multiple energetic materials and additives to improve stability or achieve certain product properties (e.g., plasticity). Examples include well-known explosive products, such as C4 (RDX combined with plasticizers, binders, and mineral oil) and Semtex (a mixture of RDX and PETN with several additives including plasticizers, binders, antioxidants, and sometimes a coloring agent).

**Figure 7:**
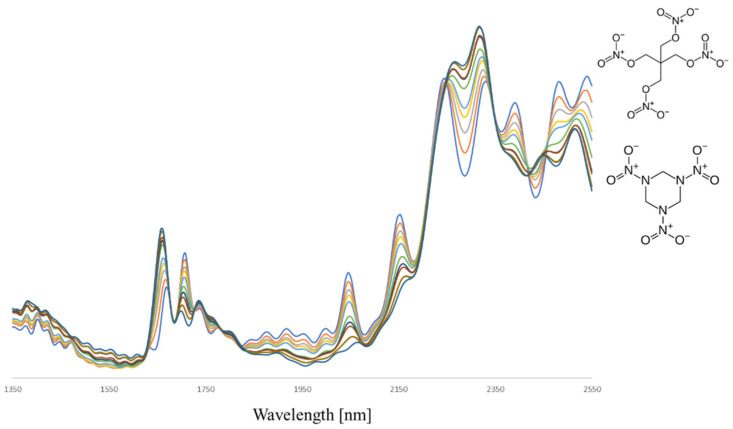
SNV-corrected, average NIR spectra (n = 5) of binary mixtures of RDX and PETN in different weight ratios (from 100% RDX to 100% PETN in steps of 10 wt%).

To investigate whether explosive mixtures can be correctly characterized using portable NIR, binary mixtures of RDX and PETN at different ratios were prepared and analyzed at TNO. The observed spectra, as shown in Figure 7, illustrate the composite nature with spectral features of both energetic compounds. Due to the high spectral reproducibility for a given composition, a gradual transition from the pure compound spectrum of RDX to the PETN reference spectrum (and vice versa) is observed.

**Figure 8:**
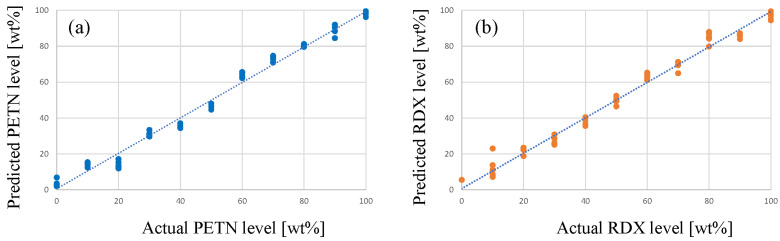
PLS model (seven principal components) performance for binary mixtures of PETN (**a**) and RDX (**b**). The regression line is created from the full mixture data set (n = 55, 5 spectra per composition, 11 compositions), whereas the individual markers show the random cross-validation (20 segments of 2–3 random samples) results for samples not included in the calibration.

Such transitions provide opportunities for quantitative analysis through multivariate regression methods, such as principal component regression (PCR) or partial least squares (PLS). With the use of spectral data from calibration samples of known composition (such as those depicted in Figure 7), the level of RDX and PETN in case samples can be estimated using a PLS model. Figure 8 shows the random cross-validation results of the PLS model for binary RDX/PETN mixtures, the associated RMSEP (root mean square error of prediction) is below 4 wt%.

However, the cross-validation process only involves binary mixtures and thus excludes potential systematic errors due to the presence of additional minor components in forensic case samples. To investigate the sensitivity of the model to small spectral contributions not related to the explosive compounds, the PETN and RDX level was estimated for several Semtex and C4 samples in the collection of TNO. The NIR spectra of these so-called ‘plastic explosives’ (hand-moldable, solid product formulations used for mining and military purposes), as shown in Figure S3 of the Supplemental Information, clearly show a PETN- or RDX-like signature indicative of a high level of PETN, RDX, or mixtures thereof. The results obtained with the PLS and LDA-NAS (discussed in more detail in Section 3.5) models, as provided in Table S3 (Supplemental Information), were unfortunately not consistent enough for a trustworthy semi-quantitative analysis. Depending on the model applied, the spectral contribution from the additives (plasticizers, binders, preservatives, and colorants) and the product properties (a dense moldable solid as compared to the powder standards and mixtures) led to significant systematic errors. For more accurate quantitation, tailor-made reference samples need to be prepared that better represent these products. However, the results clearly show that rapid NIR screening provides a robust and trustworthy qualitative analysis to distinguish real plastic explosives from hoax materials such as clay.

Smokeless powders (SPs) represent another interesting product class consisting of a rather complex mixture of organic energetic compounds and additives with nitrocellulose as the main propellant. Additional energetic ingredients include nitroglycerine (double-base formulations) and nitroguanidine (triple-base products). In addition, SPs contain several non-energetic substances such as ethyl or methyl centralite, dimethyl- of dibutyl-phthalate, dinitro-toluene (burn rate regulators), di-phenylamine, and 2- or 4-nitro-diphenylamine (stabilizers). SP NIR spectra as depicted in Figure S4 of the Supplemental Information clearly bear resemblance to the spectral features of nitrocellulose. However, it should be noted that not all SPs in the sample set could be characterized with NIR, most darkly colored products exhibit excessive absorption of the incident radiation from the NIR ‘light’ source. This extensive absorption leads to loss of compound specific details in the spectrum as is also illustrated in Figure S4. For the SPs only 11 of the 32 products analyzed yielded a NIR spectrum that was sufficiently characteristic for the LDA-NAS model (described in more detail in Section 3.5) to consistently report the presence of nitrocellulose. This, unfortunately, indicates limited applicability of NIR spectroscopy for the characterization of smokeless powder products.

### 3.4. NIR Analysis of Inorganic Oxidizers and Pyrotechnic Mixtures

**Figure 9:**
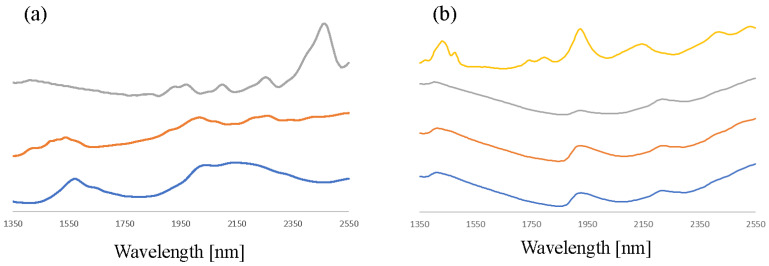
SNV-corrected, average NIR spectra (n = 5, n = 25 for KClO_3_ and KClO_4_) of (**a**) KNO_3_, urea nitrate, and ammonium nitrate (grey, orange, and blue trace, respectively) and (**b**) NaClO_4_ (monohydrate), NaClO_3_, KClO_4_, and KClO_3_ (yellow, grey, orange, and blue trace, respectively). The offset has been adjusted to facilitate spectrum comparison.

In forensic explosive analysis, a very diverse set of substances, both of organic and inorganic nature, are of interest and need to be identified. Like Raman spectroscopy, NIR can also provide useful spectral data for the identification and differentiation of salts. However, absolute absorption values are typically limited leading to a reduced signal-to-noise ratio. Furthermore, as is illustrated in Figure 9, the spectra are less detailed compared to those obtained for organic explosives, which can make it more difficult to identify the correct raw material. This especially seems to be the case for NaClO_3_, KClO_3_, and KClO_4_, for which the non-informative spectra seem visually very similar. Interestingly, the NIR signal for the NaClO_4_ standard used in this study stood out as being significantly more detailed and different from the other chlorate and perchlorate salts. The reason for this is that this standard contains water of crystallization (sodium perchlorate monohydrate); this phenomenon has also been reported for salts of MDMA and heroin [40]. Although water of crystallization is not known for potassium nitrate, the NIR spectra of the nitrate salts in Figure 9 show sufficient detail and difference for unambiguous identification of the nitrate salt type.

**Figure 10:**
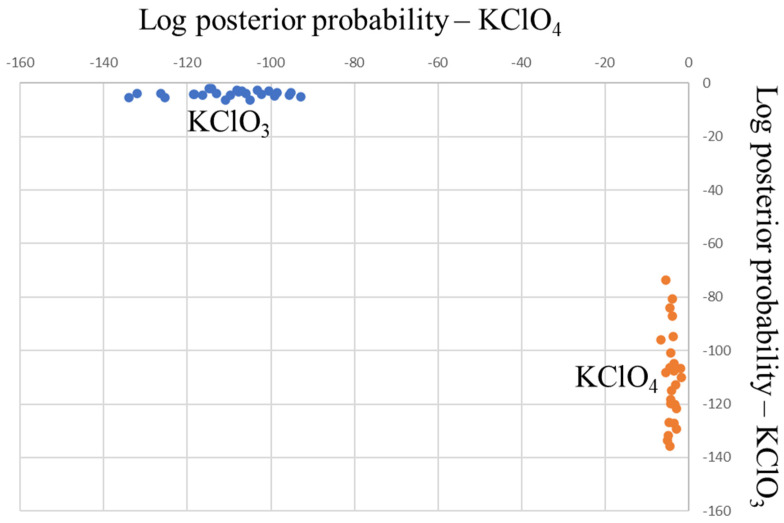
LDA discrimination plot (KClO_4_ assignment vs. KClO_3_ assignment) for the potassium chlorate and perchlorate NIR calibration data (5 samples, 5 spectra per sample, 25 spectra in total each). The full separation of the KClO_4_ and KClO_3_ clusters (orange and blue dots, respectively) indicate 100% model accuracy.

Although from visual inspection the differentiation of perchlorate salts seems to be difficult, supervised multivariate data analysis methods, such as Linear Discriminant Analysis or PLS-DA (Partial Least Squares-Discriminant Analysis), might be able to successfully exploit minimal spectral differences to facilitate a correct chemical identification. Figure 10 depicts the LDA discrimination plot for the differentiation of KClO_4_ and KClO_3_ (NIR spectra given in Figure 9b, orange and blue trace, respectively). Data pre-processing involved a Savitzky–Golay first derivative after SNV. PCA was employed to reduce the dimension of the dataset and in total, seven principal components (accounting for 97% of the data variation) were used to build the LDA model. For the current data set (25 spectra in total, 5 samples and 5 spectra per sample for both KClO_3_ and KClO_4_), a 100% correct classification rate was achieved (it should be noted that no external test samples have been included in the validation, hence the performance could be lower under casework conditions). This shows that by using a sub-selection of reference data in combination with a dedicated chemometric model, additional information can be extracted to assist chemical identification. In this case, a two-stage approach could be used, first discovering the presence of a salt of the chlorate/perchlorate class on the basis of general spectral features, followed by the application of a powerful supervised multivariate data analysis method to establish the exact species (chlorate or perchlorate, potassium or sodium).

Inorganic substances are typically not used as energetic material as such, but act as oxidizers and need to be mixed with fuels. Frequently encountered pyrotechnic mixtures include flash powder (a mixture of typically 70 wt% KClO_4_ and 30 wt% aluminum powder) and black powder (containing KNO_3_, sulfur, and charcoal). Unfortunately, for these two important types of pyrotechnic mixtures, the recording of informative NIR spectra was hampered by material properties. For black powder, the issue is related to the full absorption of the incident NIR electromagnetic radiation across the entire wavelength range. As a result, insufficient refracted photons reach the detector to record a detailed spectrum. In terms of absorbance, the signal is very high and lacks spectral detail to detect the presence of KNO_3_. For flash powder, the reverse situation seems to exist as the presence of very fine aluminum powder causes extensive elastic reflection. This results in a very high fraction of the incident NIR ‘light’ being directed back to the detector, translating in a very low absorbance value that hampers the identification of KClO_4_. Typical, uninformative black powder and flash powder NIR spectra are shown in Figure S5 of the Supplemental Information. These adverse phenomena could potentially be circumvented by modifying the measurement configuration and this is a topic for further study.

### 3.5. NIR-Based Identification of Energetic Materials in Forensic Casework

As a final demonstration of the potential of portable NIR for the rapid, robust, and on-scene identification of intact explosives, a set of anonymized forensic case samples from the Netherlands Forensic Institute (NFI) was analyzed. These samples were acquired as part of criminal investigations and are thus fully realistic and representative of the chemical complexity and diversity encountered in forensic practice. The advanced data analysis framework recently developed by TIPb data scientists to identify drugs of abuse in street samples [38] was used as a starting point to develop a tailor-made data analysis interface for NIR-based forensic explosive analysis. This involved the creation of a matrix library (Table S2, Supplemental Information), the construction of an LDA model for compound selection, and the creation of a NAS model to match a composite spectrum to the actual NIR data for an unknown sample.

The results of the NIR analysis of a total of 58 NFI case samples (290 scans) and 60 samples (300 scans) from the negative sample set (Section 2.3) are summarized in Table 1. All individual results at sample and scan levels can be found in Tables S4 and S5 of the Supplemental Information. The NIR analysis of the negative samples with the advanced data model confirms the findings reported in the previous sections; the NIR spectra of the energetic materials, specifically the organic explosives, are highly characteristic and consequently other materials will not easily yield a spectrum that is very similar. In this study, this resulted in a 100% TNR (true-negative rate), or in other words, a 0% FPR (false-positive rate). This means that if the NIR analyzer reports the presence of an energetic material, this information is highly robust and trustworthy and possibly even admissible in court. In a crime scene setting, it means that security measures are not taken in vain after a positive test result. However, when looking closer at the results of the NFI case samples, it becomes clear that the same does not hold for the true-positive/false-negative (TPR/FNR) rates. For ETN, HMTD, PETN, TNT, and Tetryl, a 100% correct identification score was obtained. However, for TATP, RDX, AN, and NC, in total 11 false-negative results were recorded, leading to an overall TPR of 81% (FNR = 19%). Although this sample set is too small to draw definite conclusions, the relatively low TPR does flag a challenge in forensic explosive investigation in comparison to the chemical identification of drugs of abuse. As the chemical state of the casework samples is more diverse, this can lead to false-negative results for samples that are strongly contaminated, wet, show extensive degradation, or are part of unknown formulations (e.g., Vitezit product formulations containing AN). This, of course, hampers the practical implementation of the technique, since false-negative results can affect the outcome of a court case, but more importantly can lead to serious safety incidents on scene when bomb disposal experts wrongly assume that an explosive device does not pose a threat on the basis of a negative NIR test result. It should be noted that this issue is not specific to NIR; any portable analysis technique will be challenged when faced with strongly contaminated or degraded energetic materials. However, this challenge is specific to forensic explosive analysis and needs to be adequately addressed as will be discussed in more detail in the Discussion and Conclusions section.

## 4. Discussion and Conclusions

The results presented in this work convincingly demonstrate the added value of portable NIR for the rapid, robust chemical identification of explosives and energetic raw materials, either at the incident scene, in a police station, or in a forensic laboratory setting. NIR spectra of organic explosives of the nitro-aromatic, nitro-amine, nitro-ester, and peroxide class, are highly selective and rich in compound specific details. Therefore, chemically very similar compounds of interest, such as ETN/PETN and RDX/HMX, can easily be differentiated on the basis of a NIR spectrum that can be acquired in a few seconds. This detailed chemical information in the spectra can also be exploited to detect energetic materials in more complex product formulations, such as plastic explosives. However, spectral contributions from unknown additives can hamper quantitative analysis with multi-variate regression techniques like PLS. In addition, NIR is also applicable to most inorganic energetic materials (mostly oxidizers such as ammonium nitrate and potassium perchlorate). However, for inorganic compounds, the spectra tend to be less characteristic and hence powerful supervised chemometric methods such as LDA are required to distinguish, for instance, chlorate and perchlorate salts. The excellent chemical selectivity provided by NIR in the 1350–2550 nm wavelength range was demonstrated with a 0% false-positive rate when analyzing a substantial set of known negatives, including food products, household chemicals, known hoax materials, drugs of abuse, and raw materials used to produce explosives. However, for a representative set of forensic casework samples that reflect the actual state and quality of the evidence materials, a substantial false-negative rate was obtained. Excessive degradation of aged material, poor quality of home-made explosives, residual water in insufficiently dried products, and contamination due to poor synthesis conditions are known to experts and the resulting variation in the NIR spectra is typically not accounted for in a pure compound reference library. Model performance in terms of false-negative rate could possibly be improved by including a range of spectra for each compound in the reference matrix on the basis of forensic casework.

Given these promising findings, a relevant question is what additional steps are needed to successfully introduce portable NIR in forensic, law enforcement, bomb disposal, and security practice. Additionally, could the methodology be so robust that additional confirmatory analyses are not required and that NIR analysis could, in principle, provide admissible forensic evidence? Essentially, two important developments are required to achieve large-scale implementation. Firstly, the overall data analysis framework has to be expanded, refined, optimized, and maintained. This includes spectral libraries covering the casework formulation space, application of tailor-made data analysis modules, and the optimization of decision thresholds for reporting positive or negative outcomes. To be flawless, a certain degree of inconclusive outcomes needs to be accepted; for these samples, additional chemical analysis will be necessary.

Secondly, a professional data infrastructure is needed, in which multiple portable NIR units are operated within a given organization. In such a framework, NIR data as measured on-scene is securely transferred to a cloud environment. Data preprocessing, chemometric modeling, quality control, interpretation, reporting, and archiving are all performed automatically and centrally in an analyzer network. An important prerequisite is that individual NIR analyzers are interchangeable, i.e., the analysis of a sample on different units should yield almost identical spectra. This is necessary for maintaining a common matrix and enabling centralized data analysis. In this respect, it should be noted that the current study was performed with a single portable NIR instrument. Although NIR spectrometry is known for excellent reproducibility and repeatability, it cannot be automatically assumed that the current findings translate to a NIR analyzer network. Forensic scientists of the University of Lausanne recently introduced a NIR analyzer network for illicit drug identification and quantification [41]. Convincing results were presented with the use of the handheld MicroNIR instrument that operates in the 950–1650 nm range in combination with an app on a smartphone to receive and transfer measurement data and receive the results from the cloud environment. These developments show that ‘on-scene NIR analysis as a service’ is not an abstract concept but is rapidly becoming a reality [42].

## Figures and Tables

**Table 1:** Confusion matrix showing portable NIR analyzer performance for forensic explosives casework samples from the NFI (excluding the TATP/HMTD mixtures and AN emulsions because of deviating sample and product properties) and a collected set of ‘true-negative’ samples; green = correct result (NIR analysis matches material composition), yellow = false negative (NIR analysis does not show the presence of an energetic material in the sample).

Confusion Matrix		Powder Puck NIR Result									
		ETN	TATP	HMTD	PETN	RDX	TNT	Tetryl	AN	NC	Neg
Sample Identity	ETN	2/2									
	TATP		1/3								2/3
	HMTD			9/9							
	PETN				8/8						
	RDX					3/4					1/4
	TNT						5/5				
	Tetryl							10/10			
	AN								7/13		6/13
	NC									2/4	2/4
	Neg										60/60

## Data Availability

The data presented in this study are available on request from the corresponding author. The data are not publicly available for security reasons (risk of abuse).

## Supplementary Materials

The following supporting information can be downloaded at: https://www.mdpi.com/article/10.3390/s23083804/s1, Figure S1: SNV-corrected, average NIR spectra (n = 5) of (a) PETN and ETN (orange and blue trace, respectively) and (b) RDX and HMX (orange and blue trace, respectively). Spectral details in the 1350–1625 nm range are shown. The offset has been adjusted to facilitate spectrum comparison. Figure S2: SNV-corrected, average NIR spectra (n = 5) of binary mixtures of RDX and PETN in different weight ratios. The offset has been adjusted to stack the NIR spectra.; Figure S3: Typical NIR spectra PETN-RDX based plastic explosives from the collection of TNO.; Figure S4: SNV-corrected, average NIR spectra (n = 5) of (a) SP SRN395, SP SRN357, and dry NC (grey, orange, and blue trace, respectively) and (b) SP SRN394, SP SRN306, and SP SRN330 (grey, orange, and blu trace, respectively). The offset has been adjusted to facilitate spectrum comparison. For the SP products in panel (a) the LDA-NAS model reported the presence of NC whereas for the SP products in panel (b) the results were inconclusive (false negative).; Figure S5: Typical NIR signals observed for (a) black powder and (b) flash powder samples (colors indicate different samples).; Table S1: Additional information TNO samples; Table S2: Explosives matrix for NIR-based identification of explosives in the case work samples using the 3-stage TIPb model; Table S3: Model predictions and product information of plastic explosives from the collection of TNO; Table S4: Results of the Powder Puck analysis (individual scans) of the NFI case work samples; Table S5: Results of the Powder Puck analysis (individual scans) of the samples from the negatives set.

## References

[1] Almog J., Zitrin S., Marshall M., Oxley J. (2009). Colorimetric Detection of Explosives. Aspects of Explosives Detection.

[2] Pesenti A., Taudte R.V., McCord B., Doble P., Roux C., Blanes L. (2014). Coupling Paper-Based Microfluidics and Lab on a Chip Technologies for Confirmatory Analysis of Trinitro Aromatic Explosives. Anal. Chem..

[3] Peters K.L., Corbin I., Kaufman L.M., Zreibe K., Blanes L., McCord B.R. (2015). Simultaneous colorimetric detection of improvised explosive compounds using microfluidic paper-based analytical devices (μPADs). Anal. Methods.

[4] Ewing R.G., Atkinson D.A., Eiceman G.A., Ewing G.J. (2001). A critical review of Ion Mobility Spectrometry for the detection of explosives and explosive related compounds. Talanta.

[5] Madhusudhan P., Latha M.M. (2013). Ion Mobility Spectrometry for the detection of explosives. Int. J. Eng. Res. Technol..

[6] Burns D., Mathias S., McCullough B.J., Hopley C.J., Douce D., Lumley N., Bajic S., Sears P. (2022). Ambient Ionisation Mass spectrometry for the trace detection of explosives using a Portable Mass Spectrometer. Int. J. Mass Spectrom..

[7] Li Z., Askim J.R., Suslick S. (2019). The Optoelectronic Nose: Colorimetric and Fluorometric Sensor Arrays. Chem. Rev..

[8] Bolse N., Eckstein R., Habermehl A., Hernandez-Sosa G., Eschenbaum C., Lemmer U. (2017). Reliability of Aerosol Jet Printed Fluorescence Quenching Sensor Arrays for the Identification and Quantification of Explosive Vapors. ACS Omega.

[9] Chuvashov R.D., Verbitskiy E.V., Baranova A.A., Khoklov K.O., Shulgin B.V. (2019). Investigation of Novel Substrates for Fluorescent Sensors to Identification of Nitroaromatic Compounds. AIP Conf. Proc..

[10] Woodka M.D., Schnee V.P. (2010). Flurorescent Polymer Sensor Array for Detection and Discrimination of Explosives in Water. Anal. Chem..

[11] Xin Y., Wang Q., Liu T., Wang L., Li J., Fang Y. (2012). A portable and autonomous multichannel fluorescence detector for on-line and in situ explosive detection in aquous phase. Lab Chip.

[12] Sun X., Wang Y., Lei Y. (2015). Fluorescence based Explosives Detection: From Mechanisms to Sensory Materials. Chem. Soc. Rev..

[13] Peveler W.J., Roldan A., Hollingsworth N., Porter M.J., Parkin I.P. (2016). Multichannel Detection and Differentiation of Explosives with a Quantum Dot Array. ACS Nano.

[14] Aznar-Gadea E., Rodrguez-Canto P.J., Sánchez S.A., Martínez-Pastor J.P., Abargues R. (2022). Luminescent CdSe Quantum Dot Arrays for Rapid Sensing of Explosive Taggants. ACS App. Nano Mater..

[15] Yu H.A., DeTata D.A., Lewis S.W., Silvester D.S. (2017). Recent developments in the electrochemical detection of explosives: Towards field-deployable devices for forensic science. TrAC.

[16] Hay C.E., Lee J., Silvester D.B. (2020). A methodology to detect explosive residues using a gelled ionic liquid-based field-deployable electrochemical device. J. Electroanal. Chem..

[17] Arman A., Sağlam Ş., Üzer A., Apak R. (2022). Electrochemical determination of nitroaromatic explosives using glassy carbon/multi-walled carbon nanotube/polyethyleneimine electrode coated with nanoparticles. Talanta.

[18] Apak R., Üzer A., Sağlam Ş., Arman A. (2022). Selective electrochemical detection of explosives with nanomaterial-based electrodes. Electroanalysis.

[19] Benson S., Speers N., Otieno-Alego V., Beveridge A. (2011). Portable explosive detection instruments. Forensic Investigation of Explosions.

[20] Harvey S., Peters T.J., Wright B.W. (2003). Safety considerations for sample analysis using a Near-Infrared (785 nm) Raman laser source. Appl. Spectrosc..

[21] Kranenburg R.F., Verduin J., Weesepoel Y., Alewijn M., Heerschop M., Koomen G., Keizers P., Bakker F., Wallace F., van Esch A. (2020). Rapid and robust on-scene detection of cocaine in street samples using a handheld Near-Infrared spectrometer and machine learning algorithms. Drug Test Anal..

[22] Ozaki Y., Morisawa Y., Ozaki Y., Huck C., Tsuchikawa S., Engelsen S.B. (2021). Principles and Characteristics of NIR spectroscopy. Near-Infrared Spectroscopy.

[23] Small G.W. (2006). Chemometrics and Near-Infrared Spectroscopy: Avoiding the pitfalls. TrAC.

[24] Canal C.M., Saleem A., Green R.J., Hutchins D.A. (2010). Near-infrared spectroscopy for personal screening. Proc. SPIE.

[25] de la Ossa M.A.F., Amigo J.M., García-Ruiz C. (2014). Detection of residues from explosive manipulation by near-infrared hyperspectral imaging: A promising forensic tool. Forensic Sci. Int..

[26] de la Ossa M.A.F., García-Ruiz C., Amigo J.M. (2014). Near-infrared spectral imaging for the analysis of dynamite residues on human handprints. Talanta.

[27] Li D., Wang A., Li Y., Cui F., Wu J., Cao J., Cao Z., Wang Y., Qiao Y. (2021). Application of NIR spectroscopy in explosive powder surface contamination remote detection. Spectrosc. Spect. Anal..

[28] Mattos E.C., Moreira E.D. (2004). Determination of the HMX and RDX content in synthesized energetic material by HPLC, FT-MIR, and FT-NIR spectroscopies. Quim. Nova.

[29] Zou Q., Deng G., Guo X., Jiang W., Li F. (2013). A green analytical tool for in-process determination of RDX content of propellant using the NIR system. ACS Sustain. Chem. Eng..

[30] Su P., Liang W., Zhang G., Wen X., Chang H., Meng Z., Xue M., Qiu L. (2021). Quantitative detection of components in polymer-bonded explosives through near-infrared spectroscopy with partial least square regression. ACS Omega.

[31] Itozaki H. (2020). Near-infrared inspection technology of bottled explosive liquid in airports. NIR News.

[32] Risoluti R., Gregori A., Schiavone S., Materazzi S. (2018). “Click and screen” technology for the detection of explosives on human hands by a portable microNIR-chemometrics platform. Anal. Chem..

[33] Grammatikaki A.M., Raptakis A., Gounaridis L., Athanasopoulos A., Gounaridis D., Groumas P., Dadoukis A., Maltezos E., Karagiannidis L., Ouzounoglou E. (2022). Portable FT-NIR spectroscopic sensor for detection of chemical precursors of explosives using advanced prediction algorithms. Proc. SPIE.

[34] Beć K.B., Grabska J., Siesler H.W., Huck C.W. (2020). Handheld near-infrared spectrometers: Where are we heading?. NIR News.

[35] Beć K.B., Grabska J., Huck C.W. (2021). Principles and applications of miniaturized near-infrared (NIR) spectrometers. Chem. Eur. J.

[36] Kranenburg R.F., Ramaker H.J., Sap S., van Asten A.C. (2022). A Calibration Friendly Approach to Identify Drugs of Abuse Mixtures with a Portable Near-Infrared Analyzer. Drug Test Anal..

[37] Kranenburg R.F., Weesepoel Y., Alewijn M., Sap S., Arisz P.W.F., van Esch A., Keizers P.H.J., van Asten A.C. (2022). The importance of wavelength selection in on-scene identification of drugs of abuse with portable near-infrared spectroscopy. Forensic Chem..

[38] Kranenburg R.F., Ramaker H.-J., van Asten A.C. (2022). On-site forensic analysis of colored seized materials: Detection of brown heroin and MDMA-tablets by a portable NIR spectrometer. Drug Test. Anal..

[39] Kranenburg R.F., Ramaker H.-J., van Asten A.C. (2022). Portable near-infrared spectroscopy for the isomeric differentiation of new psychoactive substances. Forensic Sci. Int..

[40] Kranenburg R.F., Ramaker H.-J., Weesepoel Y., Arisz P.W.F., Keizers P.H.J., van Esch A., Zieltjens-van Uzem C., van den Berg J.D.J., Hulshof J.W., Bakels S. (2023). The influence of water of crystallization in NIR-based MDMA·HCl detection. Forensic Chem..

[41] Coppey F., Bécue A., Sacré P.-Y., Ziemons E.M., Hubert P., Esseiva P. (2020). Providing illicit drugs results in five seconds using ultra-portable NIR technology: An opportunity for forensic laboratories to cope with the trend toward the decentralization of forensic capabilities. Forensic Sci. Int..

[42] NIRLAB Homepage. https://nirlab.com/.

