# Supplementary material for: Rapid and On-Scene Chemical Identification of Intact Explosives with Portable Near-Infrared Spectroscopy and Multivariate Data Analysis
Source: Sensors (Basel). 2023 Apr 7;23(8):3804. doi: 10.3390/s23083804 (PMC10146942; doi:10.3390/s23083804)
Supplement: Supplementary file 1 [file sensors-23-03804-s001.zip › sensors-2299471-supplementary.pdf]

Supplemental Information
for
Rapid and on-scene chemical identification of intact explosives with portable Near Infrared Spectroscopy and multivariate data analysis

Irene M. van Damme[1,2*], Pol Mestres-Fitó[1], Henk-Jan Ramaker[3], Annemieke Hulsbergen[2], Antoine E.D.M. van der Heijden[4], Ruben F. Kranenburg[1,5], Arian C. van Asten[1,2,6]

[1] Van 't Hoff Institute for Molecular Sciences, University of Amsterdam, Science Park 904, Amsterdam 1098 XH, The Netherlands
[2] Netherlands Forensic Institute (NFI), Laan van Ypenburg 6, Den Haag 2497 GB, The Netherlands
[3] TIPb, Koningin Wilhelminaplein 30, Amsterdam 1062 KR, The Netherlands
[4] TNO Defence, Safety and Security, Dept. Energetic Materials, Ypenburgse Boslaan 2, 2496 ZA, The Hague, The Netherlands
[5] Dutch National Police, Unit Amsterdam, Forensic Laboratory, Kabelweg 25, Amsterdam 1014 BA, The Netherlands
[6] Co van Ledden Hulsebosch Center (CLHC), Amsterdam Center for Forensic Science and Medicine, Science Park 904, Amsterdam 1098 XH, The Netherlands
[*] Corresponding author. *E-mail address:* i.m.vandamme@uva.nl (I.M. van Damme).

__Contents__

Table S1. Additional information TNO samples
Table S2. Explosives matrix for NIR-based identification of explosives in the case work samples using the 3-stage TIPb model
Figure S1. SNV-corrected, average NIR spectra (n=5) of a) PETN and ETN (orange and blue trace, respectively) and b) RDX and HMX (orange and blue trace, respectively). Spectral details in the 1350-1625 nm range are shown. The offset has been adjusted to facilitate spectrum comparison.
Figure S2. SNV-corrected, average NIR spectra (n=5) of binary mixtures of RDX and PETN in different weight ratios. The offset has been adjusted to stack the NIR spectra.
Figure S3. Typical NIR spectra PETN-RDX based plastic explosives from the collection of TNO.
Table S3. Model predictions and product information of plastic explosives from the collection of TNO
Figure S4. SNV-corrected, average NIR spectra (n=5) of a) SP SRN395, SP SRN357, and dry NC (grey, orange, and blue trace, respectively) and b) SP SRN394, SP SRN306, and SP SRN330 (grey, orange, and blue trace, respectively). The offset has been adjusted to facilitate spectrum comparison. For the SP products in panel a) the LDA-NAS model reported the presence of NC whereas for the SP products in panel b) the results were inconclusive (false negative). More information can be found in the SP database: https://www.ilrc.ucf.edu/powders/index.php).
Figure S5. Typical NIR signals observed for a) black powder and b) flash powder samples (colors indicate different samples).
Table S4. Results of the Powder Puck analysis (individual scans) of the NFI case work samples
Table S5. Results of the Powder Puck analysis (individual scans) of the samples from the negatives set

Table S1. Additional information TNO samples

Explosive	Lot / batch nr.	Provider	Remarks
AN	TL 644/09	unknown	-
HMTD	-	Prepared at TNO	-
PA	TL 882/14	unknown	-
Tetryl	TL 1000/19	unknown	-
G12	TL 946/17	unknown	Black gunpowder
SFG12	TL 947/17	unknown	Sulphur free black gunpowder
ETN	-	Prepared at TNO	-
ETN	-	Prepared at TNO	-
PETN	TL 859/13	unknown	-
PETN	TL 977/18	unknown	-
TATP	-	Prepared at TNO	-
TATP	-	Prepared at TNO	-
TNT	TL 916/15	unknown	-
TNT	TL 140/96	unknown	-
TNT	TL 743/11	unknown	
C4	TL 409/06	unknown	-
C4	TL 136/02	unknown	-
DBP	TL 1104/20	unknown	M7 Type II Paste
DBP	20EM0072	unknown	8120
DBP	20EM0050	unknown	8098
NC	20043	unknown	NC 58 wet
NC	20043	unknown	NC 58, dry
NC	PM38 36_14	unknown	NC 64, wet
NC	PM38 36_14	unknown	NC 64, dry
Semtex	TL 909/15	unknown	Semtex 1A
Semtex	TL 11~~2~~23/21	unknown	Semtex 10
Semtex	TL 908/15	unknown	Semtex 10SE
Semtex	TL 911/15	unknown	Semtex 1H
Semtex	TL 910/15	unknown	Semtex C4
HMX	TL 219/03	unknown	Class 5
HMX	TL 410/06	unknown	Class 5
HMX	TL 450/06	Dyno Nobel (Norway)	Class 1
HMX	TL 485/06	Dyno Nobel (Norway)	Class 5
HMX	TL 464/07	Eurenco SA (Sorgues, France)	Class 1
HMX	TL 465/07	Eurenco SA (Sorgues, France)	Class 5
RDX	TL 105/02	SNPE (France)	Insensitive RDX, class 1 (ca. 150 micron)
RDX	TL 106/02	SNPE (France)	Insensitive RDX, class 5 (ca. 12-23 micron)
RDX	TL 107/02	SNPE (France)	Standard RDX, class 1 (ca. 150 micron)
RDX	TL 108/02	SNPE (France)	Standard RDX, class 5 (ca. 12-23 micron)
RDX	TL 125/02	Dyno Nobel (Norway)	Insensitive RDX, class 1 (ca. 150 micron)
RDX	TL 292/04	Dyno Nobel (Norway)	-
RDX	TL 133/96	unknown	ca. 2-10 micron
RDX	TL 144/96	unknown	ca. 70-150 micron
RDX	TL 168/97	unknown	ca. 250 micron
RDX	TL 170/97y	unknown	Class E (ca. 20 micron)
RDX	TL 169/97aY	unknown	ca. 300 micron
RDX	TL 959/17	unknown	-
RDX	TL 984/18	unknown	-

Table S2. Explosives and energetic raw materials reference matrix for the LDA-NAS model

1	RDX	Organic
2	HMX	Organic
3	TNT	Organic
4	Tetryl	Organic
5	Picric Acid	Organic
6	Nitrocellulose Dry	Organic
7	Nitrocellulose Wet	Organic
8	PETN	Organic
9	ETN	Organic
10	TATP Dry	Organic
11	TATP Wet	Organic
12	HMTD	Organic
13	Ammonium Nitrate	Inorganic

Figure S1. SNV-corrected, average NIR spectra (n=5) of a) PETN and ETN (orange and blue trace, respectively) and b) RDX and HMX (orange and blue trace, respectively). Spectral details in the 1350-1625 nm range are shown. The offset has been adjusted to facilitate spectrum comparison.

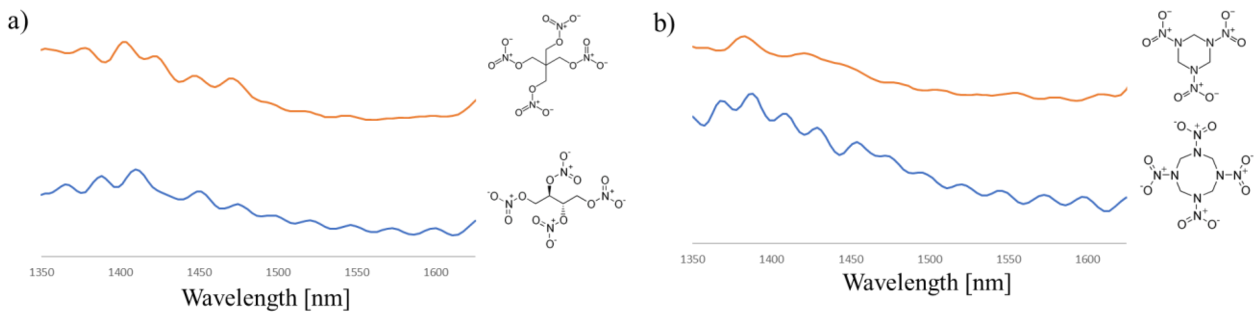

Figure S2. SNV-corrected, average NIR spectra (n=5) of binary mixtures of RDX and PETN in different weight ratios (from 100% RDX to 100% PETN in steps of 20 wt%).

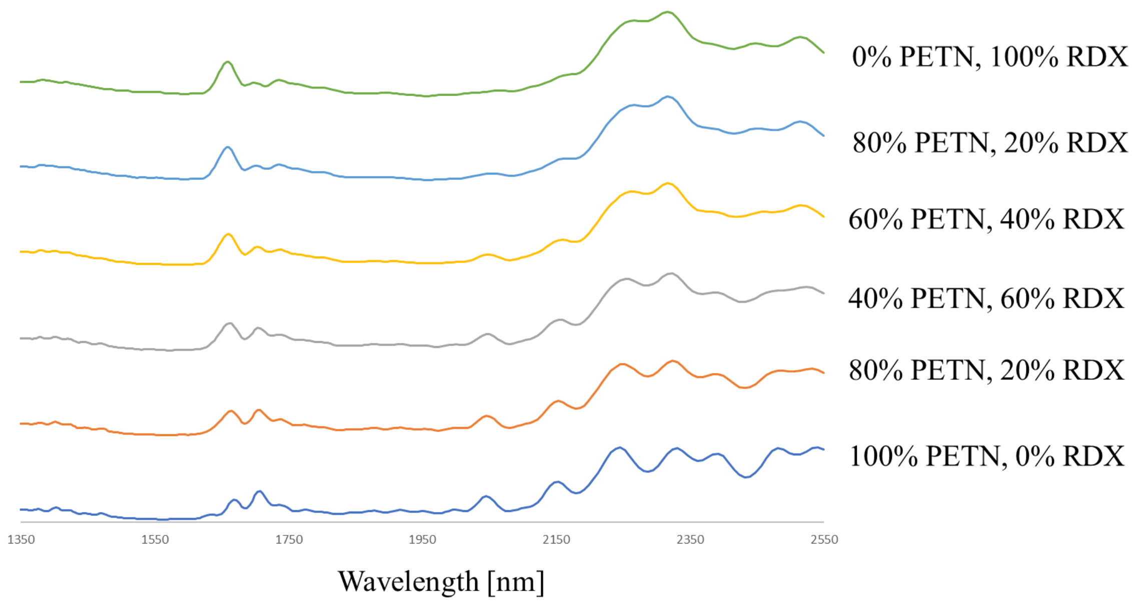

Figure S3. Typical NIR spectra of PETN-RDX based plastic explosives from the collection of TNO

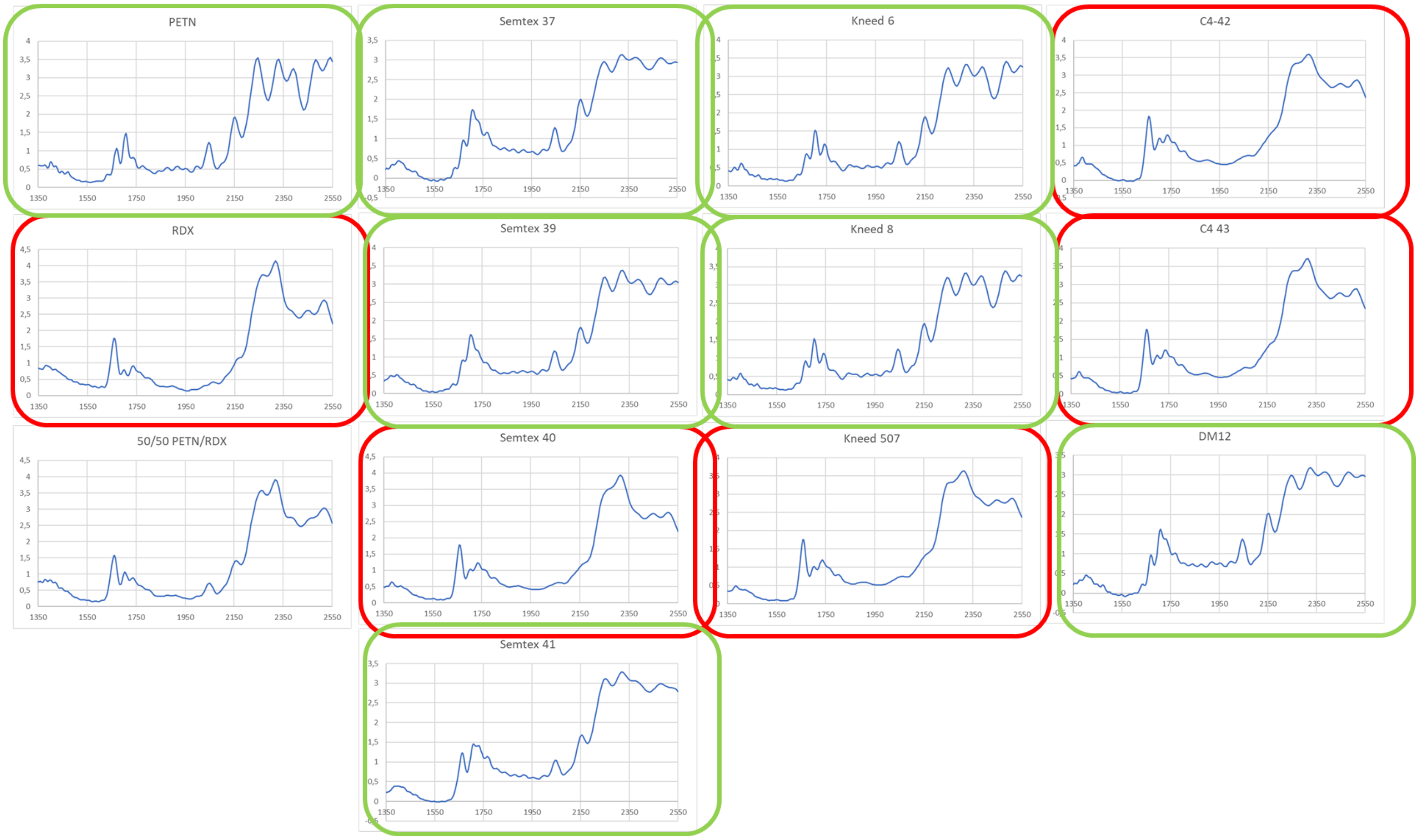

Table S3. PLS and LDA-NAS predictions (average value from 5 scans) of RDX/PETN-based plastic explosives from the TNO collection. Typical spectra (labelled as PETN-like or RDX like) can be found in Figure S1.

		**Product info**		**PLS model**		**LDA-NAS model**	
**Sample ID**	**NIR spectrum**	**PETN [wt%]**	**RDX [wt%]**	**PETN [wt%]**	**RDX [wt%]**	**PETN [wt%]**	**RDX [wt%]**
Semtex 37	PETN-like	83	0	135	-35	36	-
Semtex 39	PETN-like	78	0	108	-8	58	-
Semtex 40	RDX-like	~41	~41	46	54	-	75
Semtex 41	PETN-like	0	~90	107	-7	32	38
DM12	PETN-like	85	0	133	-33	44	-#
Kneed 6	PETN-like	86	0	101	-1	64	-
Kneed 8	PETN-like	86	0	106	-6	63	-
Kneed 507	RDX-like	0	90	67	33	-	66
C4 - 42	RDX-like	0	~90	52	48	-	83*
C4 - 43	RDX-like	0	~90	62	38	-	80

For 1 of the spectra also the presence of 17% RDX was reported

* For 4 of the 5 spectra also the presence of 12% HMX was reported

Figure S4. SNV-corrected, average NIR spectra (n=5) of a) SP SRN395, SP SRN357, and dry NC (grey, orange, and blue trace, respectively) and b) SP SRN394, SP SRN306, and SP SRN330 (grey, orange, and blue trace, respectively). The offset has been adjusted to facilitate spectrum comparison. For the SP products in panel a) the LDA-NAS model reported the presence of NC whereas for the SP products in panel b) the results were inconclusive (false negative). The SP samples originate from the Smokeless Powders Database of the National Center for Forensic Science of the University of Central Florida (Orlando, USA), more information on the products can be retrieved by searching the SRN number in the on-line SP database: https://www.ilrc.ucf.edu/powders/index.php).

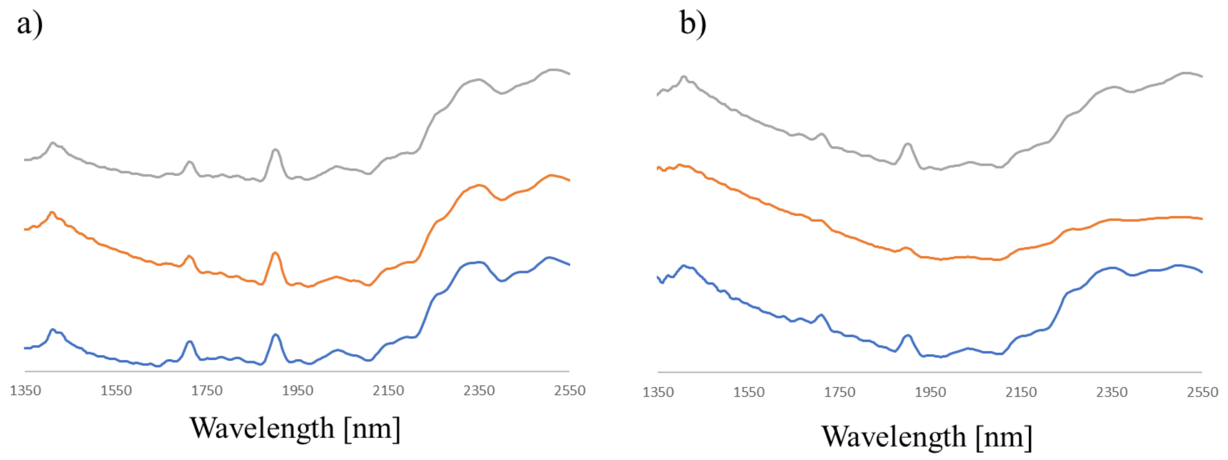

Figure S5. Typical NIR signals observed for a) black powder and b) flash powder samples (colors indicate different samples).

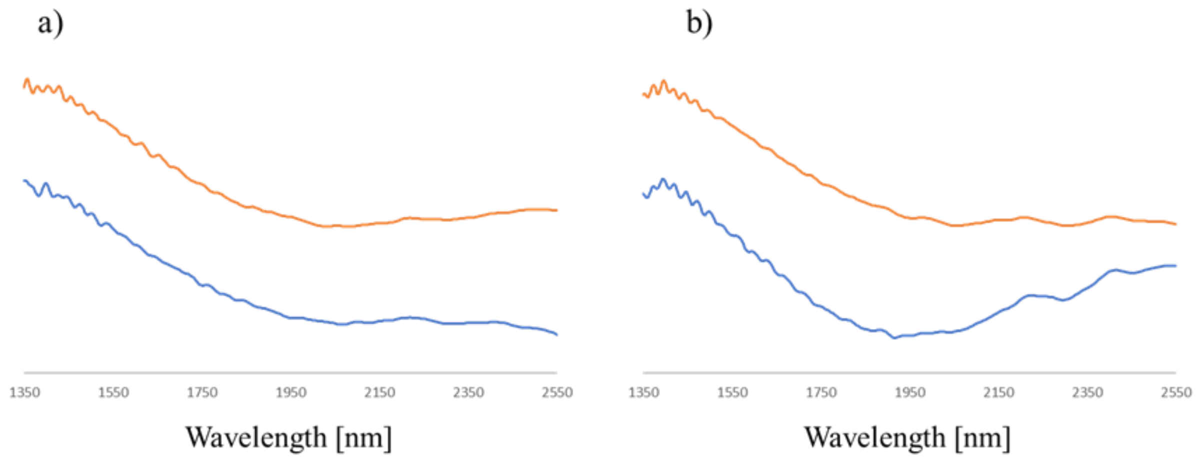

Table S4. Results of the Powder Puck analysis of the NFI case work samples
(Result of individual scans, 5 scans per sample)

**Forensic case sample**	**Power Puck ID**	**Similarity**	**Result**
ETN (White Powder)	ETN (96%)	0.96	true positive
ETN (White Powder)	ETN (79%)	0.98	true positive
ETN (White Powder)	ETN (94%)	0.94	true positive
ETN (White Powder)	ETN (99%)	0.99	true positive
ETN (White Powder)	ETN (75%)	0.97	true positive
ETN (White Powder/Contaminated)	ETN (64%)	0.93	true positive
ETN (White Powder/Contaminated)	ETN (65%)	0.93	true positive
ETN (White Powder/Contaminated)	ETN (63%)	0.92	true positive
ETN (White Powder/Contaminated)	ETN (64%)	0.93	true positive
ETN (White Powder/Contaminated)	ETN (62%)	0.92	true positive
TATP (White - grey Paste/With IPA)	Sample not part of model population - no results	0.70	false negative
TATP (White - grey Paste/With IPA)	Sample not part of model population - no results	0.66	false negative
TATP (White - grey Paste/With IPA)	Sample not part of model population - no results	0.67	false negative
TATP (White - grey Paste/With IPA)	Sample not part of model population - no results	0.66	false negative
TATP (White - grey Paste/With IPA)	Sample not part of model population - no results	0.65	false negative
TATP (White - grey Paste/With IPA)	TATP Dry (19%)	0.71	false negative
TATP (White - grey Paste/With IPA)	Sample not part of model population - no results	0.68	false negative
TATP (White - grey Paste/With IPA)	Sample not part of model population - no results	0.64	false negative
TATP (White - grey Paste/With IPA)	Sample not part of model population - no results	0.70	false negative
TATP (White - grey Paste/With IPA)	Sample not part of model population - no results	0.68	false negative
TATP (White Powder)	TATP Dry (92%)	0.92	true positive
TATP (White Powder)	TATP Dry (63%)	0.92	true positive
TATP (White Powder)	Sample not part of model population - no results	0.37	false negative
TATP (White Powder)	TATP Dry (64%)	0.90	true positive
TATP (White Powder)	Sample not part of model population - no results	0.34	false negative
HMTD (White Powder/Prepared at the NFI)	HMTD (63%)	0.90	true positive
HMTD (White Powder/Prepared at the NFI)	HMTD (62%)	0.91	true positive
HMTD (White Powder/Prepared at the NFI)	HMTD (62%)	0.91	true positive
HMTD (White Powder/Prepared at the NFI)	HMTD (59%)	0.90	true positive
HMTD (White Powder/Prepared at the NFI)	HMTD (62%)	0.90	true positive
HMTD (White Powder)	HMTD (67%)	0.91	true positive
HMTD (White Powder)	HMTD (70%)	0.93	true positive

HMTD (White Powder)	HMTD (71%)	0.92	true positive
HMTD (White Powder)	HMTD (71%)	0.92	true positive
HMTD (White Powder)	HMTD (67%)	0.93	true positive
HMTD (White Powder)	HMTD (69%)	0.92	true positive
HMTD (White Powder)	HMTD (67%)	0.92	true positive
HMTD (White Powder)	HMTD (68%)	0.92	true positive
HMTD (White Powder)	HMTD (68%)	0.93	true positive
HMTD (White Powder)	HMTD (67%)	0.91	true positive
HMTD (White Powder)	HMTD (69%)	0.93	true positive
HMTD (White Powder)	HMTD (69%)	0.94	true positive
HMTD (White Powder)	HMTD (71%)	0.94	true positive
HMTD (White Powder)	HMTD (71%)	0.94	true positive
HMTD (White Powder)	HMTD (68%)	0.94	true positive
HMTD (Yellowish Powder)	HMTD (77%)	0.95	true positive
HMTD (Yellowish Powder)	HMTD (77%)	0.95	true positive
HMTD (Yellowish Powder)	HMTD (95%)	0.95	true positive
HMTD (Yellowish Powder)	HMTD (76%)	0.95	true positive
HMTD (Yellowish Powder)	HMTD (75%)	0.95	true positive
HMTD (Yellowish Powder)	HMTD (77%)	0.95	true positive
HMTD (Yellowish Powder)	HMTD (74%)	0.94	true positive
HMTD (Yellowish Powder)	HMTD (77%)	0.95	true positive
HMTD (Yellowish Powder)	HMTD (75%)	0.95	true positive
HMTD (Yellowish Powder)	HMTD (77%)	0.94	true positive
HMTD (Yellowish Powder)	HMTD (75%)	0.94	true positive
HMTD (Yellowish Powder)	HMTD (76%)	0.95	true positive
HMTD (Yellowish Powder)	HMTD (95%)	0.95	true positive
HMTD (Yellowish Powder)	HMTD (76%)	0.95	true positive
HMTD (Yellowish Powder)	HMTD (95%)	0.95	true positive
HMTD (Yellowish Powder)	HMTD (73%)	0.94	true positive
HMTD (Yellowish Powder)	HMTD (95%)	0.95	true positive
HMTD (Yellowish Powder)	HMTD (95%)	0.95	true positive
HMTD (Yellowish Powder)	HMTD (73%)	0.93	true positive
HMTD (Yellowish Powder)	HMTD (74%)	0.93	true positive
HMTD (White Powder)	HMTD (66%)	0.90	true positive
HMTD (White Powder)	HMTD (89%)	0.89	true positive
HMTD (White Powder)	HMTD (59%)	0.90	true positive
HMTD (White Powder)	HMTD (53%)	0.87	true positive
HMTD (White Powder)	HMTD (64%)	0.90	true positive
TATP/HMTD (White Powder)	HMTD (29%)	0.70	false negative
TATP/HMTD (White Powder)	Sample not part of model population - no results	0.65	false negative
TATP/HMTD (White Powder)	HMTD (31%)	0.73	false negative
TATP/HMTD (White Powder)	HMTD (30%)	0.71	false negative

TATP/HMTD (White Powder)	HMTD (34%)	0.74	false negative
TATP/HMTD (White Powder)	HMTD (33%)	0.77	false negative
TATP/HMTD (White Powder)	HMTD (34%)	0.74	true positive
TATP/HMTD (White Powder)	HMTD (40%)	0.83	true positive
TATP/HMTD (White Powder)	HMTD (38%)	0.83	true positive
TATP/HMTD (White Powder)	HMTD (37%)	0.79	true positive
TATP/HMTD (White - grey Powder)	TATP Wet (49%) + HMTD (20%)	0.89	true positive
TATP/HMTD (White - grey Powder)	TATP Dry (61%)	0.89	true positive
TATP/HMTD (White - grey Powder)	TATP Dry (58%)	0.87	true positive
TATP/HMTD (White - grey Powder)	TATP Dry (53%) + HMTD (21%)	0.93	true positive
TATP/HMTD (White - grey Powder)	TATP Dry (59%)	0.88	true positive
TATP/HMTD (White Powder/With impurities)	HMTD (34%)	0.81	true positive
TATP/HMTD (White Powder/With impurities)	HMTD (28%)	0.75	false negative
TATP/HMTD (White Powder/With impurities)	HMTD (24%)	0.77	false negative
TATP/HMTD (White Powder/With impurities)	HMTD (47%) + TATP Wet (16%)	0.87	true positive
TATP/HMTD (White Powder/With impurities)	HMTD (33%)	0.78	false negative
PETN (Brown Paste)	PETN (44%)	0.92	true positive
PETN (Brown Paste)	PETN (44%)	0.91	true positive
PETN (Brown Paste)	PETN (39%)	0.91	true positive
PETN (Brown Paste)	PETN (42%)	0.91	true positive
PETN (Brown Paste)	PETN (43%)	0.92	true positive
PETN (Pink Paste)	PETN (65%)	0.92	true positive
PETN (Pink Paste)	PETN (64%)	0.92	true positive
PETN (Pink Paste)	PETN (67%)	0.92	true positive
PETN (Pink Paste)	PETN (62%)	0.92	true positive
PETN (Pink Paste)	PETN (65%)	0.92	true positive
PETN (White Powder)	PETN (73%)	0.96	true positive
PETN (White Powder)	PETN (75%)	0.96	true positive
PETN (White Powder)	PETN (69%)	0.95	true positive
PETN (White Powder)	PETN (73%)	0.96	true positive
PETN (White Powder)	PETN (72%)	0.96	true positive
PETN (Brown Paste)	PETN (24%)	0.85	true positive
PETN (Brown Paste)	PETN (15%)	0.82	true positive
PETN (Brown Paste)	PETN (22%)	0.84	true positive
PETN (Brown Paste)	PETN (15%)	0.82	true positive
PETN (Brown Paste)	PETN (20%)	0.84	true positive
PETN (Yellow Powder)	PETN (75%)	0.96	true positive
PETN (Yellow Powder)	PETN (75%) + RDX (12%)	0.96	true positive
PETN (Yellow Powder)	PETN (74%) + RDX (13%)	0.97	true positive

PETN (Yellow Powder)	PETN (71%)	0.96	true positive
PETN (Yellow Powder)	PETN (73%) + RDX (14%)	0.96	true positive
PETN (Brown Paste)	PETN (45%)	0.90	true positive
PETN (Brown Paste)	PETN (59%) + RDX (18%)	0.94	true positive
PETN (Brown Paste)	PETN (37%)	0.88	true positive
PETN (Brown Paste)	PETN (55%) + RDX (17%)	0.92	true positive
PETN (Brown Paste)	PETN (34%)	0.88	true positive
PETN (Yellow Paste)	PETN (57%)	0.93	true positive
PETN (Yellow Paste)	PETN (68%)	0.95	true positive
PETN (Yellow Paste)	PETN (62%) + RDX (16%)	0.94	true positive
PETN (Yellow Paste)	PETN (94%)	0.94	true positive
PETN (Yellow Paste)	PETN (55%) + RDX (18%)	0.92	true positive
PETN (Yellow - brown Paste)	PETN (55%) + RDX (18%)	0.93	true positive
PETN (Yellow - brown Paste)	PETN (58%) + RDX (18%)	0.93	true positive
PETN (Yellow - brown Paste)	PETN (94%)	0.94	true positive
PETN (Yellow - brown Paste)	PETN (51%)	0.92	true positive
PETN (Yellow - brown Paste)	PETN (37%)	0.88	true positive
RDX (White Paste)	RDX (76%)	0.96	true positive
RDX (White Paste)	RDX (77%)	0.96	true positive
RDX (White Paste)	RDX (76%)	0.96	true positive
RDX (White Paste)	RDX (97%)	0.97	true positive
RDX (White Paste)	RDX (74%)	0.95	true positive
RDX (Green Paste)	RDX (28%)	0.80	true positive
RDX (Green Paste)	RDX (30%)	0.80	true positive
RDX (Green Paste)	RDX (30%)	0.79	false negative
RDX (Green Paste)	RDX (29%)	0.79	false negative
RDX (Green Paste)	RDX (28%)	0.78	false negative
RDX (White Paste)	RDX (98%)	0.98	true positive
RDX (White Paste)	RDX (82%)	0.99	true positive
RDX (White Paste)	RDX (96%)	0.96	true positive
RDX (White Paste)	RDX (78%)	0.98	true positive
RDX (White Paste)	RDX (73%)	0.97	true positive
RDX (White Paste/With impurities)	RDX (99%)	0.99	true positive
RDX (White Paste/With impurities)	RDX (77%)	0.98	true positive
RDX (White Paste/With impurities)	RDX (74%)	0.98	true positive
RDX (White Paste/With impurities)	RDX (82%)	0.98	true positive
RDX (White Paste/With impurities)	RDX (98%)	0.98	true positive
TNT (Yellowish Paste)	TNT (93%)	1	true positive
TNT (Yellowish Paste)	TNT (100%)	1	true positive
TNT (Yellowish Paste)	TNT (100%)	1	true positive
TNT (Yellowish Paste)	TNT (92%)	1	true positive
TNT (Yellowish Paste)	TNT (92%)	1	true positive
TNT (Brownish Big pieces)	TNT (99%)	0.99	true positive

TNT (Brownish Big pieces)	TNT (99%)	0.99	true positive
TNT (Brownish Big pieces)	TNT (100%)	1	true positive
TNT (Brownish Big pieces)	TNT (100%)	1	true positive
TNT (Brownish Big pieces)	TNT (100%)	1	true positive
TNT (Brownish Powder)	TNT (98%)	0.98	true positive
TNT (Brownish Powder)	TNT (96%)	0.96	true positive
TNT (Brownish Powder)	TNT (99%)	0.99	true positive
TNT (Brownish Powder)	TNT (98%)	0.98	true positive
TNT (Brownish Powder)	TNT (73%)	0.95	true positive
TNT (Brownish Powder)	TNT (96%)	0.96	true positive
TNT (Brownish Powder)	TNT (96%)	0.96	true positive
TNT (Brownish Powder)	TNT (96%)	0.96	true positive
TNT (Brownish Powder)	TNT (96%)	0.96	true positive
TNT (Brownish Powder)	TNT (96%)	0.96	true positive
TNT (Yellow - brown Small flakes)	TNT (92%)	1	true positive
TNT (Yellow - brown Small flakes)	TNT (100%)	1	true positive
TNT (Yellow - brown Small flakes)	TNT (100%)	1	true positive
TNT (Yellow - brown Small flakes)	TNT (100%)	1	true positive
TNT (Yellow - brown Small flakes)	TNT (100%)	1	true positive
Tetryl (Grey Small stones)	Tetryl (91%)	0.91	true positive
Tetryl (Grey Small stones)	Tetryl (92%)	0.92	true positive
Tetryl (Grey Small stones)	Tetryl (91%)	0.91	true positive
Tetryl (Grey Small stones)	Tetryl (89%)	0.89	true positive
Tetryl (Grey Small stones)	Tetryl (89%)	0.89	true positive
Tetryl (Grey Powder)	Tetryl (93%)	0.93	true positive
Tetryl (Grey Powder)	Tetryl (95%)	0.95	true positive
Tetryl (Grey Powder)	Tetryl (94%)	0.94	true positive
Tetryl (Grey Powder)	Tetryl (91%)	0.91	true positive
Tetryl (Grey Powder)	Tetryl (92%)	0.92	true positive
Tetryl (Grey Powder)	Tetryl (92%)	0.92	true positive
Tetryl (Grey Powder)	Tetryl (95%)	0.95	true positive
Tetryl (Grey Powder)	Tetryl (91%)	0.91	true positive
Tetryl (Grey Powder)	Tetryl (95%)	0.95	true positive
Tetryl (Grey Powder)	Tetryl (94%)	0.94	true positive
Tetryl (Grey Powder)	Tetryl (93%)	0.93	true positive
Tetryl (Grey Powder)	Tetryl (94%)	0.94	true positive
Tetryl (Grey Powder)	Tetryl (94%)	0.94	true positive
Tetryl (Grey Powder)	Tetryl (94%)	0.94	true positive
Tetryl (Grey Powder)	Tetryl (92%)	0.92	true positive
Tetryl (Grey Powder)	Tetryl (92%)	0.92	true positive
Tetryl (Grey Powder)	Tetryl (94%)	0.94	true positive
Tetryl (Grey Powder)	Tetryl (92%)	0.92	true positive
Tetryl (Grey Powder)	Tetryl (94%)	0.94	true positive

Tetryl (Grey Powder)	Tetryl (95%)	0.95	true positive
Tetryl (Grey Powder)	Tetryl (92%)	0.92	true positive
Tetryl (Grey Powder)	Tetryl (91%)	0.91	true positive
Tetryl (Grey Powder)	Tetryl (93%)	0.93	true positive
Tetryl (Grey Powder)	Tetryl (95%)	0.95	true positive
Tetryl (Grey Powder)	Tetryl (93%)	0.93	true positive
Tetryl (Grey Powder)	Tetryl (89%)	0.89	true positive
Tetryl (Grey Powder)	Tetryl (92%)	0.92	true positive
Tetryl (Grey Powder)	Tetryl (97%)	0.97	true positive
Tetryl (Grey Powder)	Tetryl (93%)	0.93	true positive
Tetryl (Grey Powder)	Tetryl (95%)	0.95	true positive
Tetryl (Grey Powder)	Tetryl (94%)	0.94	true positive
Tetryl (Grey Powder)	Tetryl (91%)	0.91	true positive
Tetryl (Grey Powder)	Tetryl (94%)	0.94	true positive
Tetryl (Grey Powder)	Tetryl (66%) + Picric Acid (21%)	0.87	true positive
Tetryl (Grey Powder)	Tetryl (90%)	0.90	true positive
Tetryl (Grey Powder)	Tetryl (93%)	0.93	true positive
Tetryl (Grey Powder)	Tetryl (92%)	0.92	true positive
Tetryl (Grey Powder)	Tetryl (94%)	0.94	true positive
Tetryl (Grey Powder)	Tetryl (94%)	0.94	true positive
Tetryl (Grey Powder)	Tetryl (89%)	0.89	true positive
Tetryl (Grey Powder)	Tetryl (93%)	0.93	true positive
Tetryl (Grey Powder)	Tetryl (94%)	0.94	true positive
Tetryl (Grey Powder)	Tetryl (89%)	0.89	true positive
Tetryl (Grey Powder)	Tetryl (91%)	0.91	true positive
Tetryl (Grey Powder)	Tetryl (94%)	0.94	true positive
Ureumnitrate (White Powder)	Sample not part of model population - no results	0	true negative
Ureumnitrate (White Powder)	Sample not part of model population - no results	0	true negative
Ureumnitrate (White Powder)	Sample not part of model population - no results	0	true negative
Ureumnitrate (White Powder)	Sample not part of model population - no results	0	true negative
Ureumnitrate (White Powder)	Sample not part of model population - no results	0	true negative
Ammonium nitrate (White - grey Powder/Dirty)	Ammonium Nitrate (94%)	0.94	true positive
Ammonium nitrate (White - grey Powder/Dirty)	Ammonium Nitrate (66%)	0.94	true positive
Ammonium nitrate (White - grey Powder/Dirty)	Ammonium Nitrate (62%)	0.93	true positive
Ammonium nitrate (White - grey Powder/Dirty)	Ammonium Nitrate (66%)	0.93	true positive
Ammonium nitrate (White - grey Powder/Dirty)	Ammonium Nitrate (60%)	0.91	true positive

Ammonium nitrate (White - grey Powder/Dirty)	Ammonium Nitrate (66%)	0.92	true positive
Ammonium nitrate (White - grey Powder/Dirty)	Ammonium Nitrate (65%)	0.93	true positive
Ammonium nitrate (White - grey Powder/Dirty)	Ammonium Nitrate (68%)	0.93	true positive
Ammonium nitrate (White - grey Powder/Dirty)	Ammonium Nitrate (67%)	0.94	true positive
Ammonium nitrate (White - grey Powder/Dirty)	Ammonium Nitrate (67%)	0.94	true positive
Ammonium nitrate (White - yellow Powder/Dirty and wet)	Sample not part of model population - no results	0.57	false negative
Ammonium nitrate (White - yellow Powder/Dirty and wet)	Sample not part of model population - no results	0	false negative
Ammonium nitrate (White - yellow Powder/Dirty and wet)	Sample not part of model population - no results	0.54	false negative
Ammonium nitrate (White - yellow Powder/Dirty and wet)	Sample not part of model population - no results	0.49	false negative
Ammonium nitrate (White - yellow Powder/Dirty and wet)	Sample not part of model population - no results	0.52	false negative
Ammonium nitrate (White - yellow Powder/Dirty and wet)	Sample not part of model population - no results	0	false negative
Ammonium nitrate (White - yellow Powder/Dirty and wet)	Sample not part of model population - no results	0	false negative
Ammonium nitrate (White - yellow Powder/Dirty and wet)	Sample not part of model population - no results	0.70	false negative
Ammonium nitrate (White - yellow Powder/Dirty and wet)	Sample not part of model population - no results	0	false negative
Ammonium nitrate (White - yellow Powder/Dirty and wet)	Sample not part of model population - no results	0.29	false negative
Ammonium nitrate (White - yellow Powder /Dirty and wet)	Sample not part of model population - no results	0.33	false negative
Ammonium nitrate (White - yellow Powder /Dirty and wet)	Sample not part of model population - no results	0.29	false negative
Ammonium nitrate (White - yellow Powder /Dirty and wet)	Sample not part of model population - no results	0.29	false negative
Ammonium nitrate (White - yellow Powder /Dirty and wet)	Sample not part of model population - no results	0.25	false negative
Ammonium nitrate (White - yellow Powder /Dirty and wet)	Sample not part of model population - no results	0.57	false negative
Ammonium nitrate (White - brown Powder/Dirty and wet)	Sample not part of model population - no results	0.69	false negative
Ammonium nitrate (White - brown Powder/Dirty and wet)	Sample not part of model population - no results	0	false negative
Ammonium nitrate (White - brown Powder/Dirty and wet)	Sample not part of model population - no results	0	false negative
Ammonium nitrate (White - brown Powder/Dirty and wet)	Sample not part of model population - no results	0.23	false negative
Ammonium nitrate (White - brown Powder/Dirty and wet)	Sample not part of model population - no results	0	false negative
Ammonium nitrate (Yellowish Powder/Dirty)	Ammonium Nitrate (52%)	0.88	true positive
Ammonium nitrate (Yellowish Powder/Dirty)	Ammonium Nitrate (57%)	0.90	true positive
Ammonium nitrate (Yellowish Powder/Dirty)	Ammonium Nitrate (51%)	0.89	true positive
Ammonium nitrate (Yellowish Powder/Dirty)	Ammonium Nitrate (52%)	0.89	true positive

Ammonium nitrate (Yellowish Powder/Dirty)	Ammonium Nitrate (53%)	0.89	true positive
Ammonium nitrate (White Powder/A bit dirty)	Ammonium Nitrate (55%)	0.90	true positive
Ammonium nitrate (White Powder/A bit dirty)	Ammonium Nitrate (57%)	0.90	true positive
Ammonium nitrate (White Powder/A bit dirty)	Ammonium Nitrate (54%)	0.89	true positive
Ammonium nitrate (White Powder/A bit dirty)	Ammonium Nitrate (55%)	0.90	true positive
Ammonium nitrate (White Powder/A bit dirty)	Ammonium Nitrate (55%)	0.90	true positive
Ammonium nitrate (White Powder/Looks quite pure)	Ammonium Nitrate (54%)	0.90	true positive
Ammonium nitrate (White Powder/Looks quite pure)	Ammonium Nitrate (45%)	0.85	true positive
Ammonium nitrate (White Powder/Looks quite pure)	Ammonium Nitrate (52%)	0.88	true positive
Ammonium nitrate (White Powder/Looks quite pure)	Ammonium Nitrate (50%)	0.88	true positive
Ammonium nitrate (White Powder/Looks quite pure)	Ammonium Nitrate (50%)	0.88	true positive
Ammonium nitrate (White Powder/Looks quite pure)	Ammonium Nitrate (63%)	0.91	true positive
Ammonium nitrate (White Powder/Looks quite pure)	Ammonium Nitrate (41%)	0.83	true positive
Ammonium nitrate (White Powder/Looks quite pure)	Ammonium Nitrate (62%)	0.92	true positive
Ammonium nitrate (White Powder/Looks quite pure)	Ammonium Nitrate (67%)	0.93	true positive
Ammonium nitrate (White Powder/Looks quite pure)	Ammonium Nitrate (66%)	0.93	true positive
Ammonium nitrate (White Powder/From Sigma-Aldrich)	Ammonium Nitrate (52%)	0.89	true positive
Ammonium nitrate (White Powder/From Sigma-Aldrich)	Ammonium Nitrate (23%)	0.73	false negative
Ammonium nitrate (White Powder/From Sigma-Aldrich)	Ammonium Nitrate (51%)	0.87	true positive
Ammonium nitrate (White Powder/From Sigma-Aldrich)	Ammonium Nitrate (49%)	0.88	true positive
Ammonium nitrate (White Powder/From Sigma-Aldrich)	Ammonium Nitrate (47%)	0.86	true positive
Vitezit (Red Paste)	Ammonium Nitrate (53%) + Nitrocellulose Wet (6%)	0.82	true positive
Vitezit (Red Paste)	Ammonium Nitrate (51%) + Nitrocellulose Wet (6%)	0.80	true positive
Vitezit (Red Paste)	Ammonium Nitrate (27%)	0.77	false negative
Vitezit (Red Paste)	Ammonium Nitrate (29%)	0.75	false negative
Vitezit (Red Paste)	Ammonium Nitrate (26%)	0.72	false negative
Vitezit (Red Paste)	Ammonium Nitrate (33%)	0.75	false negative
Vitezit (Red Paste)	Ammonium Nitrate (38%)	0.78	false negative
Vitezit (Red Paste)	Ammonium Nitrate (34%)	0.76	false negative
Vitezit (Red Paste)	Ammonium Nitrate (36%)	0.77	false negative
Vitezit (Red Paste)	Ammonium Nitrate (36%)	0.76	false negative
Nitrocellulose (White - yellow Powder/10 years old)	Sample not part of model population - no results	0.24	false negative

Nitrocellulose (White - yellow Powder/10 years old)	Sample not part of model population - no results	0.24	false negative
Nitrocellulose (White - yellow Powder/10 years old)	Sample not part of model population - no results	0.28	false negative
Nitrocellulose (White - yellow Powder/10 years old)	Sample not part of model population - no results	0.26	false negative
Nitrocellulose (White - yellow Powder/10 years old)	Sample not part of model population - no results	0.24	false negative
Nitrocellulose (Yellow Powder/20 years old)	Nitrocellulose Dry (58%) + Nitrocellulose Wet (32%)	0.98	true positive
Nitrocellulose (Yellow Powder/20 years old)	Nitrocellulose Dry (62%) + Nitrocellulose Wet (28%)	0.98	true positive
Nitrocellulose (Yellow Powder/20 years old)	Nitrocellulose Dry (59%) + Nitrocellulose Wet (31%)	0.98	true positive
Nitrocellulose (Yellow Powder/20 years old)	Nitrocellulose Dry (59%) + Nitrocellulose Wet (33%)	0.98	true positive
Nitrocellulose (Yellow Powder/20 years old)	Nitrocellulose Dry (60%) + Nitrocellulose Wet (31%)	0.99	true positive
Nitrocellulose (White Powder)	Sample not part of model population - no results	0.28	false negative
Nitrocellulose (White Powder)	Sample not part of model population - no results	0.28	false negative
Nitrocellulose (White Powder)	Sample not part of model population - no results	0.28	false negative
Nitrocellulose (White Powder)	Sample not part of model population - no results	0.28	false negative
Nitrocellulose (White Powder)	Sample not part of model population - no results	0.28	false negative
Nitrocellulose (Yellow Small cylinders/Small cylinders)	Nitrocellulose Dry (83%)	0.99	true positive
Nitrocellulose (Yellow Small cylinders/Small cylinders)	Nitrocellulose Dry (98%)	0.98	true positive
Nitrocellulose (Yellow Small cylinders/Small cylinders)	Nitrocellulose Dry (99%)	0.99	true positive
Nitrocellulose (Yellow Small cylinders/Small cylinders)	Nitrocellulose Dry (99%)	0.99	true positive
Nitrocellulose (Yellow Small cylinders/Small cylinders)	Nitrocellulose Dry (99%)	0.99	true positive

Table S5. Results of the Powder Puck analysis (individual scans) of the samples from the negatives set (Result of individual scans, 5 scans per sample)

**Sample**	**Power Puck ID**	**Similarity**
3-MMC	Sample not part of model population - no results	0.25
3-MMC	Sample not part of model population - no results	0.21
3-MMC	Sample not part of model population - no results	0.24
3-MMC	Sample not part of model population - no results	0.24
3-MMC	Sample not part of model population - no results	0.25
Amphetamine (1)	Sample not part of model population - no results	0.47
Amphetamine (1)	Sample not part of model population - no results	0.46
Amphetamine (1)	Sample not part of model population - no results	0.49
Amphetamine (1)	Sample not part of model population - no results	0.48
Amphetamine (1)	Sample not part of model population - no results	0.49
Amphetamine (2)	Sample not part of model population - no results	0.46
Amphetamine (2)	Sample not part of model population - no results	0.46
Amphetamine (2)	Sample not part of model population - no results	0.47
Amphetamine (2)	Sample not part of model population - no results	0.44
Amphetamine (2)	Sample not part of model population - no results	0.47
Aspirine	Sample not part of model population - no results	0.00
Aspirine	Sample not part of model population - no results	0.00
Aspirine	Sample not part of model population - no results	0.00
Aspirine	Sample not part of model population - no results	0.00
Aspirine	Sample not part of model population - no results	0.00
Benzocaine	Sample not part of model population - no results	0.00
Benzocaine	Sample not part of model population - no results	0.00
Benzocaine	Sample not part of model population - no results	0.00
Benzocaine	Sample not part of model population - no results	0.00
Benzocaine	Sample not part of model population - no results	0.00
Boric Acid	Sample not part of model population - no results	0.00
Boric Acid	Sample not part of model population - no results	0.00
Boric Acid	Sample not part of model population - no results	0.00
Boric Acid	Sample not part of model population - no results	0.00
Boric Acid	Sample not part of model population - no results	0.00
Brown Heroin	Sample not part of model population - no results	0.55
Brown Heroin	Sample not part of model population - no results	0.54
Brown Heroin	Sample not part of model population - no results	0.53
Brown Heroin	Sample not part of model population - no results	0.54
Brown Heroin	Sample not part of model population - no results	0.53
Levamisole (1)	Sample not part of model population - no results	0.38
Levamisole (1)	Sample not part of model population - no results	0.38
Levamisole (1)	Sample not part of model population - no results	0.39
Levamisole (1)	Sample not part of model population - no results	0.36

Levamisole (1)	Sample not part of model population - no results	0.37
Cocaine HCl (1)	Sample not part of model population - no results	0.19
Cocaine HCl (1)	Sample not part of model population - no results	0.19
Cocaine HCl (1)	Sample not part of model population - no results	0.21
Cocaine HCl (1)	Sample not part of model population - no results	0.19
Cocaine HCl (1)	Sample not part of model population - no results	0.19
Cocaine HCl (1)	Sample not part of model population - no results	0.39
Cocaine HCl (1)	Sample not part of model population - no results	0.42
Cocaine HCl (1)	Sample not part of model population - no results	0.40
Cocaine HCl (1)	Sample not part of model population - no results	0.41
Cocaine HCl (1)	Sample not part of model population - no results	0.39
Cocaine Base (1)	Sample not part of model population - no results	0.18
Cocaine Base (1)	Sample not part of model population - no results	0.17
Cocaine Base (1)	Sample not part of model population - no results	0.18
Cocaine Base (1)	Sample not part of model population - no results	0.17
Cocaine Base (1)	Sample not part of model population - no results	0.17
Cocaine Base (2)	Sample not part of model population - no results	0.20
Cocaine Base (2)	Sample not part of model population - no results	0.21
Cocaine Base (2)	Sample not part of model population - no results	0.21
Cocaine Base (2)	Sample not part of model population - no results	0.22
Cocaine Base (2)	Sample not part of model population - no results	0.21
Cocaine HCl (2)	Sample not part of model population - no results	0.54
Cocaine HCl (2)	Sample not part of model population - no results	0.56
Cocaine HCl (2)	Sample not part of model population - no results	0.57
Cocaine HCl (2)	Sample not part of model population - no results	0.55
Cocaine HCl (2)	Sample not part of model population - no results	0.55
Coffeine	Sample not part of model population - no results	0.55
Coffeine	Sample not part of model population - no results	0.54
Coffeine	Sample not part of model population - no results	0.54
Coffeine	Sample not part of model population - no results	0.55
Coffeine	Sample not part of model population - no results	0.55
Diltiazem	Sample not part of model population - no results	0.00
Diltiazem	Sample not part of model population - no results	0.00
Diltiazem	Sample not part of model population - no results	0.00
Diltiazem	Sample not part of model population - no results	0.00
Diltiazem	Sample not part of model population - no results	0.00
Phenacitin	Sample not part of model population - no results	0.25
Phenacitin	Sample not part of model population - no results	0.26
Phenacitin	Sample not part of model population - no results	0.26
Phenacitin	Sample not part of model population - no results	0.25
Phenacitin	Sample not part of model population - no results	0.26
Flunitrazapam	Sample not part of model population - no results	0.00
Flunitrazapam	Sample not part of model population - no results	0.00

Flunitrazapam	Sample not part of model population - no results	0.00
Flunitrazapam	Sample not part of model population - no results	0.00
Flunitrazapam	Sample not part of model population - no results	0.00
GHB	Sample not part of model population - no results	0.52
GHB	Sample not part of model population - no results	0.52
GHB	Sample not part of model population - no results	0.52
GHB	Sample not part of model population - no results	0.52
GHB	Sample not part of model population - no results	0.51
Glucose	Sample not part of model population - no results	0.00
Glucose	Sample not part of model population - no results	0.00
Glucose	Sample not part of model population - no results	0.00
Glucose	Sample not part of model population - no results	0.00
Glucose	Sample not part of model population - no results	0.00
White Heroin	Sample not part of model population - no results	0.36
White Heroin	Sample not part of model population - no results	0.35
White Heroin	Sample not part of model population - no results	0.36
White Heroin	Sample not part of model population - no results	0.36
White Heroin	Sample not part of model population - no results	0.36
Ketamine (1)	Sample not part of model population - no results	0.51
Ketamine (1)	Sample not part of model population - no results	0.50
Ketamine (1)	Sample not part of model population - no results	0.50
Ketamine (1)	Sample not part of model population - no results	0.50
Ketamine (1)	Sample not part of model population - no results	0.50
Ketamine (2)	Sample not part of model population - no results	0.57
Ketamine (2)	Sample not part of model population - no results	0.47
Ketamine (2)	Sample not part of model population - no results	0.44
Ketamine (2)	Sample not part of model population - no results	0.57
Ketamine (2)	Sample not part of model population - no results	0.00
Ketamine (3)	Sample not part of model population - no results	0.36
Ketamine (3)	Sample not part of model population - no results	0.14
Ketamine (3)	Sample not part of model population - no results	0.16
Ketamine (3)	Sample not part of model population - no results	0.35
Ketamine (3)	Sample not part of model population - no results	0.48
Lactose	Sample not part of model population - no results	0.00
Lactose	Sample not part of model population - no results	0.00
Lactose	Sample not part of model population - no results	0.00
Lactose	Sample not part of model population - no results	0.00
Lactose	Sample not part of model population - no results	0.00
Levamisole (2)	Sample not part of model population - no results	0.53
Levamisole (2)	Sample not part of model population - no results	0.55
Levamisole (2)	Sample not part of model population - no results	0.53
Levamisole (2)	Sample not part of model population - no results	0.53
Levamisole (2)	Sample not part of model population - no results	0.54

Lidocaine	Sample not part of model population - no results	0.26
Lidocaine	Sample not part of model population - no results	0.25
Lidocaine	Sample not part of model population - no results	0.26
Lidocaine	Sample not part of model population - no results	0.26
Lidocaine	Sample not part of model population - no results	0.26
Mannitol	Sample not part of model population - no results	0.18
Mannitol	Sample not part of model population - no results	0.17
Mannitol	Sample not part of model population - no results	0.16
Mannitol	Sample not part of model population - no results	0.17
Mannitol	Sample not part of model population - no results	0.18
MDMA (1)	Sample not part of model population - no results	0.31
MDMA (1)	Sample not part of model population - no results	0.37
MDMA (1)	Sample not part of model population - no results	0.36
MDMA (1)	Sample not part of model population - no results	0.36
MDMA (1)	Sample not part of model population - no results	0.36
MDMA (2)	Sample not part of model population - no results	0.35
MDMA (2)	Sample not part of model population - no results	0.37
MDMA (2)	Sample not part of model population - no results	0.36
MDMA (2)	Sample not part of model population - no results	0.37
MDMA (2)	Sample not part of model population - no results	0.37
Mephedrone	Sample not part of model population - no results	0.46
Mephedrone	Sample not part of model population - no results	0.46
Mephedrone	Sample not part of model population - no results	0.46
Mephedrone	Sample not part of model population - no results	0.46
Mephedrone	Sample not part of model population - no results	0.45
Methamphetamine (1)	Sample not part of model population - no results	0.54
Methamphetamine (1)	Sample not part of model population - no results	0.54
Methamphetamine (1)	Sample not part of model population - no results	0.56
Methamphetamine (1)	Sample not part of model population - no results	0.55
Methamphetamine (1)	Sample not part of model population - no results	0.55
Methamphetamine (2)	Sample not part of model population - no results	0.47
Methamphetamine (2)	Sample not part of model population - no results	0.44
Methamphetamine (2)	Sample not part of model population - no results	0.46
Methamphetamine (2)	Sample not part of model population - no results	0.46
Methamphetamine (2)	Sample not part of model population - no results	0.45
Oxazepam	Sample not part of model population - no results	0.00
Oxazepam	Sample not part of model population - no results	0.00
Oxazepam	Sample not part of model population - no results	0.00
Oxazepam	Sample not part of model population - no results	0.00
Oxazepam	Sample not part of model population - no results	0.00
Paracetamol	Sample not part of model population - no results	0.50
Paracetamol	Sample not part of model population - no results	0.48
Paracetamol	Sample not part of model population - no results	0.48

Paracetamol	Sample not part of model population - no results	0.49
Paracetamol	Sample not part of model population - no results	0.50
Caffeine	Sample not part of model population - no results	0.38
Caffeine	Sample not part of model population - no results	0.39
Caffeine	Sample not part of model population - no results	0.38
Caffeine	Sample not part of model population - no results	0.39
Caffeine	Sample not part of model population - no results	0.39
Phenacitine_Caffeine_Levamisol	Sample not part of model population - no results	0.62
Phenacitine_Caffeine_Levamisol	Sample not part of model population - no results	0.62
Phenacitine_Caffeine_Levamisol	Sample not part of model population - no results	0.62
Phenacitine_Caffeine_Levamisol	Sample not part of model population - no results	0.63
Phenacitine_Caffeine_Levamisol	Sample not part of model population - no results	0.62
Procaine	Sample not part of model population - no results	0.28
Procaine	Sample not part of model population - no results	0.28
Procaine	Sample not part of model population - no results	0.26
Procaine	Sample not part of model population - no results	0.27
Procaine	Sample not part of model population - no results	0.26
Promethazine	Sample not part of model population - no results	0.55
Promethazine	Sample not part of model population - no results	0.54
Promethazine	Sample not part of model population - no results	0.55
Promethazine	Sample not part of model population - no results	0.55
Promethazine	Sample not part of model population - no results	0.54
Sildenafil	Sample not part of model population - no results	0.00
Sildenafil	Sample not part of model population - no results	0.00
Sildenafil	Sample not part of model population - no results	0.00
Sildenafil	Sample not part of model population - no results	0.00
Sildenafil	Sample not part of model population - no results	0.00
Sildenafil	Sample not part of model population - no results	0.00
THC	Sample not part of model population - no results	0.00
THC	Sample not part of model population - no results	0.00
THC	Sample not part of model population - no results	0.00
THC	Sample not part of model population - no results	0.00
THC	Sample not part of model population - no results	0.00
Vitamin C	Sample not part of model population - no results	0.00
Vitamin C	Sample not part of model population - no results	0.00
Vitamin C	Sample not part of model population - no results	0.00
Vitamin C	Sample not part of model population - no results	0.00
Vitamin C	Sample not part of model population - no results	0.00
Starch	Sample not part of model population - no results	0.19
Starch	Sample not part of model population - no results	0.18
Starch	Sample not part of model population - no results	0.20
Starch	Sample not part of model population - no results	0.17
Starch	Sample not part of model population - no results	0.17

Agar Agar	Sample not part of model population - no results	0.31
Agar Agar	Sample not part of model population - no results	0.29
Agar Agar	Sample not part of model population - no results	0.30
Agar Agar	Sample not part of model population - no results	0.31
Agar Agar	Sample not part of model population - no results	0.28
Baking Powder	Sample not part of model population - no results	0.00
Baking Powder	Sample not part of model population - no results	0.00
Baking Powder	Sample not part of model population - no results	0.00
Baking Powder	Sample not part of model population - no results	0.00
Baking Powder	Sample not part of model population - no results	0.00
Baking Soda	Sample not part of model population - no results	0.00
Baking Soda	Sample not part of model population - no results	0.00
Baking Soda	Sample not part of model population - no results	0.00
Baking Soda	Sample not part of model population - no results	0.00
Baking Soda	Sample not part of model population - no results	0.00
Coffee Creamer	Sample not part of model population - no results	0.00
Coffee Creamer	Sample not part of model population - no results	0.00
Coffee Creamer	Sample not part of model population - no results	0.00
Coffee Creamer	Sample not part of model population - no results	0.00
Coffee Creamer	Sample not part of model population - no results	0.20
Pain Killer (1)	Sample not part of model population - no results	0.22
Pain Killer (1)	Sample not part of model population - no results	0.00
Pain Killer (1)	Sample not part of model population - no results	0.00
Pain Killer (1)	Sample not part of model population - no results	0.00
Pain Killer (1)	Sample not part of model population - no results	0.22
Erythritol	Sample not part of model population - no results	0.19
Erythritol	Sample not part of model population - no results	0.15
Erythritol	Sample not part of model population - no results	0.16
Erythritol	Sample not part of model population - no results	0.19
Erythritol	Sample not part of model population - no results	0.22
Fondant	Sample not part of model population - no results	0.18
Fondant	Sample not part of model population - no results	0.21
Fondant	Sample not part of model population - no results	0.20
Fondant	Sample not part of model population - no results	0.20
Fondant	Sample not part of model population - no results	0.19
Corn starch	Sample not part of model population - no results	0.22
Corn starch	Sample not part of model population - no results	0.22
Corn starch	Sample not part of model population - no results	0.22
Corn starch	Sample not part of model population - no results	0.23
Corn starch	Sample not part of model population - no results	0.22
Playdough clay	Sample not part of model population - no results	0.30
Playdough clay	Sample not part of model population - no results	0.17
Playdough clay	Sample not part of model population - no results	0.33

Playdough clay	Sample not part of model population - no results	0.24
Playdough clay	Sample not part of model population - no results	0.31
Powdered sugar	Sample not part of model population - no results	0.33
Powdered sugar	Sample not part of model population - no results	0.31
Powdered sugar	Sample not part of model population - no results	0.32
Powdered sugar	Sample not part of model population - no results	0.31
Powdered sugar	Sample not part of model population - no results	0.31
Salt	Sample not part of model population - no results	0.00
Salt	Sample not part of model population - no results	0.00
Salt	Sample not part of model population - no results	0.00
Salt	Sample not part of model population - no results	0.00
Salt	Sample not part of model population - no results	0.00
Sugar	Sample not part of model population - no results	0.28
Sugar	Sample not part of model population - no results	0.15
Sugar	Sample not part of model population - no results	0.30
Sugar	Sample not part of model population - no results	0.29
Sugar	Sample not part of model population - no results	0.29
Vetsin (Mono-sodium glutamate)	Sample not part of model population - no results	0.00
Vetsin (Mono-sodium glutamate)	Sample not part of model population - no results	0.00
Vetsin (Mono-sodium glutamate)	Sample not part of model population - no results	0.00
Vetsin (Mono-sodium glutamate)	Sample not part of model population - no results	0.00
Vetsin (Mono-sodium glutamate)	Sample not part of model population - no results	0.00
Vetsin (Mono-sodium glutamate)	Sample not part of model population - no results	0.00
Washing detergent	Sample not part of model population - no results	0.26
Washing detergent	Sample not part of model population - no results	0.26
Washing detergent	Sample not part of model population - no results	0.27
Washing detergent	Sample not part of model population - no results	0.26
Washing detergent	Sample not part of model population - no results	0.26
Wheat flour	Sample not part of model population - no results	0.20
Wheat flour	Sample not part of model population - no results	0.22
Wheat flour	Sample not part of model population - no results	0.22
Wheat flour	Sample not part of model population - no results	0.22
Wheat flour	Sample not part of model population - no results	0.20
Pain Killer (2)	Sample not part of model population - no results	0.43
Pain Killer (2)	Sample not part of model population - no results	0.44
Pain Killer (2)	Sample not part of model population - no results	0.44
Pain Killer (2)	Sample not part of model population - no results	0.44
Pain Killer (2)	Sample not part of model population - no results	0.44
Xylitol (1)	Sample not part of model population - no results	0.00
Xylitol (1)	Sample not part of model population - no results	0.00
Xylitol (1)	Sample not part of model population - no results	0.00
Xylitol (1)	Sample not part of model population - no results	0.00
Xylitol (1)	Sample not part of model population - no results	0.00

Xylitol (2)	Sample not part of model population - no results	0.00
Xylitol (2)	Sample not part of model population - no results	0.00
Xylitol (2)	Sample not part of model population - no results	0.00
Xylitol (2)	Sample not part of model population - no results	0.00
Xylitol (2)	Sample not part of model population - no results	0.00
